# Complex interplay of riboregulatory mechanisms at the *gcvT*-NGFG_01513-*gcvH* operon of *Neisseria gonorrhoeae*

**DOI:** 10.1128/jb.00593-25

**Published:** 2026-03-05

**Authors:** Susanne Bauer, Thomas Rudel, Dagmar Beier

**Affiliations:** 1 Chair of Microbiology, Theodor-Boveri-Institute, University of Würzburg 9190, Würzburg, Germany; University of Notre Dame, Notre Dame, Indiana, USA

**Keywords:** glycine tandem riboswitch, sRNA, *Neisseria gonorrhoeae*, post-transcriptional regulation

## Abstract

The sibling sRNAs NgncR_162 and NgncR_163 are global post-transcriptional regulators of *Neisseria gonorrhoeae* metabolism, which affect amino acid uptake and biosynthesis, the citric acid and methyl citric acid cycle, and in particular pathways contributing to serine-glycine metabolism. Here, we show that *gcvT*, *gcvP,* and *gcvH,* constituting specific components of the glycine cleavage system which converts glycine to carbon dioxide, ammonia, and 5,10-methylene tetrahydrofolate, are negatively regulated by the sibling sRNAs, while the fourth component of the GCV multienzyme complex, the L-protein, which is also involved in other cellular reactions, is not targeted by NgncR_162 and NgncR_163. In contrast to *gcvP* and *gcvL*, the expression of *gcvT* and *gcvH,* being part of a tricistronic operon, is upregulated in the presence of glycine via a translational tandem glycine riboswitch. In the absence of glycine, aptamer 2 contributes to the formation of an inhibitory secondary structure that sequesters the ribosomal-binding site of *gcvT,* which notably is also targeted by the sibling sRNAs. To our knowledge, this is the first example of dual control by a riboswitch and an sRNA where the same segment of the target mRNA is involved in base-pairing interactions with both the cis- and the trans-acting riboregulator, suggesting considerable structural flexibility of the *gcvT*-NGFG_01513-*gcvH* mRNA.

**IMPORTANCE:**

In the bacterial world, riboregulation is extremely widespread and controls virtually all aspects of the life of bacterial cells. Riboregulatory mechanisms are very diverse and involve *trans*- and *cis*-acting regulatory elements such as small RNAs and riboswitches that are able to control gene expression by direct binding of small molecules. Here, we report a very peculiar and rare example of riboregulation in which an mRNA is regulated by both *trans*-acting sibling sRNAs and a glycine-responsive tandem riboswitch, which control glycine metabolism of the human pathogen *Neisseria gonorrhoeae*. This regulatory interplay of sibling sRNAs with a tandem riboswitch allows integration of different cellular signals and represents an intriguing new facet of riboregulation.

## INTRODUCTION

*Neisseria gonorrhoeae*, the causative agent of the sexually transmitted disease gonorrhea, is considered a major public health threat due to the increasing number of infections, development of resistance to every class of currently available antibiotics, and the fact that vaccines protecting against gonococcal infection are still not available (1–3). Gonococci are able to infect different host tissues and to successfully cope with both the local microbiota and the host’s immune response, and therefore are in need of effective regulatory mechanisms to ensure rapid adaptation of their gene expression profile to changing environmental conditions. Over the past two decades, non-coding RNAs have been increasingly recognized as important post-transcriptional regulators of bacterial metabolism and virulence (4–6). Trans-acting small RNAs (sRNAs) are transcribed from intergenic regions and undergo short imperfect base-pairing interactions with their target mRNAs, thereby affecting translation initiation, mRNA stability, or Rho-dependent transcription termination (7). Although more than 200 non-coding transcripts have been detected in *N. gonorrhoeae* by deep RNA sequencing (RNA-seq) (8, 9), only a few sRNAs have been characterized, including NrrF and FnrS, which are induced by iron depletion and anaerobic conditions, respectively (10–15).

Recently, we reported an RNA-seq-based approach to comprehensively identify genes under the control of the sRNAs NgncR_162 and NgncR_163. These sRNAs are encoded adjacent to each other, exhibit 78% of sequence identity, and fold into a similar secondary structure comprising three stem-loops and therefore are referred to as siblings. NgncR_162/163 is abundant when gonococci are grown in rich or chemically defined medium (16, 17); however, environmental or metabolic signals affecting their transcription are still unknown. NgncR_162/163 regulates genes belonging to different functional categories like *Neisseria*’s incomplete denitrification pathway, amino acid and carbohydrate transport, amino acid metabolism, and methyl citric acid and citric acid cycle (17). Serine/glycine metabolism was shown to be a prominent metabolic pathway targeted by NgncR_162/163 by downregulation of the glycine transporter NGFG_01721, protein H from the glycine cleavage (GCV) system, and serine hydroxymethyltransferase GlyA (17). GlyA converts serine to glycine with concomitant formation of 5,10-methylene tetrahydrofolate (5,10-mTHF). However, in gonococci, the reverse reaction might be more important, since they lack a *serA* homolog encoding 3-phosphoglycerate dehydrogenase, which catalyzes the first step in serine synthesis starting from 3-phosphoglycerate, and consequently, no labeled serine was detected in stable isotope incorporation experiments, when bacteria were fed with fully ^13^C-labeled glucose (17). The GCV system is evolutionarily conserved in mammals, plants, and bacteria and catalyzes the oxidative cleavage of glycine to carbon dioxide, ammonia, and 5,10-mTHF (18). It is a multienzyme complex formed by the proteins P, T, and H, and dihydrolipoamide dehydrogenase (L protein), which is also involved in other cellular reactions (18–20). GcvP catalyzes the pyridoxal phosphate-dependent decarboxylation of glycine (21). The remaining methylamine group is transferred to the lipoyl prosthetic group of the carrier protein GcvH. Finally, ammonia is released from the methylamine group by GcvT, and 5,10-mTHF is formed by the transfer of the remaining C_1_-unit to THF. The lipoic acid component of GcvH is reoxidized by dihydrolipoamide dehydrogenase with concomitant reduction of NAD^+^. C_1_-units generated by glycine cleavage can then be used in the synthesis of serine, thymidine, and purines (reviewed in reference [22]). Interestingly, in *Francisella tularensis,* where the GCV system is part of the serine biosynthetic pathway, deletion of *gcvT* resulted in attenuation in a murine model of pneumonic tularemia (23). In addition, a function of *Mycoplasma bovis* GcvH, which is independent of its enzymatic activity, was observed, since the protein acted as an apoptosis inhibitor by interacting with the host cell endoplasmic reticulum-resident protein kinase Brsk2 (24). In *E. coli,* P, H, and T proteins are encoded in the *gcvTHP* operon (25). However, this genomic organization is not universally conserved in bacteria. For example, in *Streptomyces griseus, gcvTH* is cotranscribed, while *gcvP* is encoded at a different gene locus (26), and a similar organization is also suggested for *N. gonorrhoeae* according to the genome annotation. The *gcvTHP* operon of *E. coli* is regulated in response to glycine by a complex interplay of three regulatory proteins, that is, GcvA, its accessory protein GcvR, and Lrp. In the absence of glycine, GcvA and GcvR assemble to a protein complex by heteromultimerization, which binds to the *gcvTHP* promoter region and forms a repression loop with the help of Lrp, which assists in DNA-bending. Glycine binding to GcvR blocks GcvA/GcvR complex formation, thereby disrupting the repression loop and allowing GcvA to act as an activator of *gcvTHP* transcription (reviewed in reference [22]). In *Pseudomonas aeruginosa,* glycine-responsive expression of the *gcs2* cluster comprising the genes encoding the GCV system, serine hydroxymethyltransferase, and serine dehydratase is accomplished by the TyrR-like enhancer binding protein GcsR (27).

Riboswitches are another well-characterized means of glycine-responsive expression control and have been found in the 5′-UTR of *gcv* genes from many bacterial species (26, 28–31). Riboswitches are non-coding RNA elements located in the 5′-UTR of bacterial mRNAs, which contain structured aptamer domains for ligand binding connected to a downstream expression platform. The binding of a specific metabolite or ion induces an alternative RNA structure, resulting in ON/OFF switching of downstream gene expression. This is typically accomplished by liberation or sequestration of sequence motifs that are part of intrinsic transcription terminators or constitute the ribosomal binding site (RBS) of the downstream gene (32, 33). Furthermore, riboswitch control can affect mRNA levels by modulating access to regulatory sequences required for Rho-dependent transcription termination or RNase E cleavage (34, 35). Translationally active riboswitches may even exert a dual function by controlling both translation initiation and mRNA levels via an indirect effect whereby inhibition of translation favors Rho-dependent transcription termination within the coding sequence (36).

Thus, in this manuscript, we provide evidence that in *N. gonorrhoeae*, the control of glycine metabolism involves a complex interplay of two RNA-based regulatory mechanisms. We show that the expression of *gcvT* and *gcvH* is induced by glycine availability via a translational tandem glycine riboswitch located in the *gcvT* 5′-UTR. Furthermore, we show that the three core components of the GCV system, *gcvT*, *gcvH,* and *gcvP*, are directly targeted by the sibling sRNAs NgncR_162 and NgncR_163 to integrate another, yet undefined signal into the expression control of the GCV system.

## RESULTS

### Glycine cleavage system genes *gcvT*, *gcvP,* and *gcvH* are targeted by the sibling sRNAs NgncR_162 and NgncR_163

Transcriptome analysis of a deletion mutant and subsequent target validation had demonstrated that genes involved in serine/glycine metabolism are controlled by the sibling sRNAs, including *gcvH* encoding the GCV system H protein (17). Therefore, we asked whether the other components of the GCV system, which did not show significant differential expression in our RNA-seq approach, might be under the control of the sibling sRNAs as well. Prediction of sRNA-target interactions using IntaRNA (37) revealed sRNA hybridization to the ribosomal-binding site in the 5′-UTR of both *gcvT* and *gcvP,* engaging the single-stranded region 1 (SSR1) (*gcvT*) and stem-loop 2 (*gcvP*) sequence of both sRNAs (Fig. S1A and B), while several putative interactions within the coding regions were predicted in the case of *gcvL* (hybridization energies ranging from −1.34 kcal/mol to −8.55 kcal/mol) (Fig. S1C). According to RT-PCR analysis (Fig. S2)*, gcvT* and *gcvP* are part of polycistronic transcription units (*gcvT*-NGFG_01513-*gcvH* and NGFG_01593-*gcvP*, with NGFG_01513 and NGFG_01593 encoding proteins of unknown function), while *gcvL* forms a monocistronic transcript (8). Another gene possibly involved in glycine metabolism, NGFG_01544 encoding M61 family glycyl aminopeptidase, which in our RNA-seq analysis was significantly downregulated in the absence of the sibling sRNAs (17), was also chosen for further target validation. IntaRNA analysis (37) predicted sRNA hybridization within the CDS also in the case of this putative target gene (Fig. S1D).

**Fig 1:**
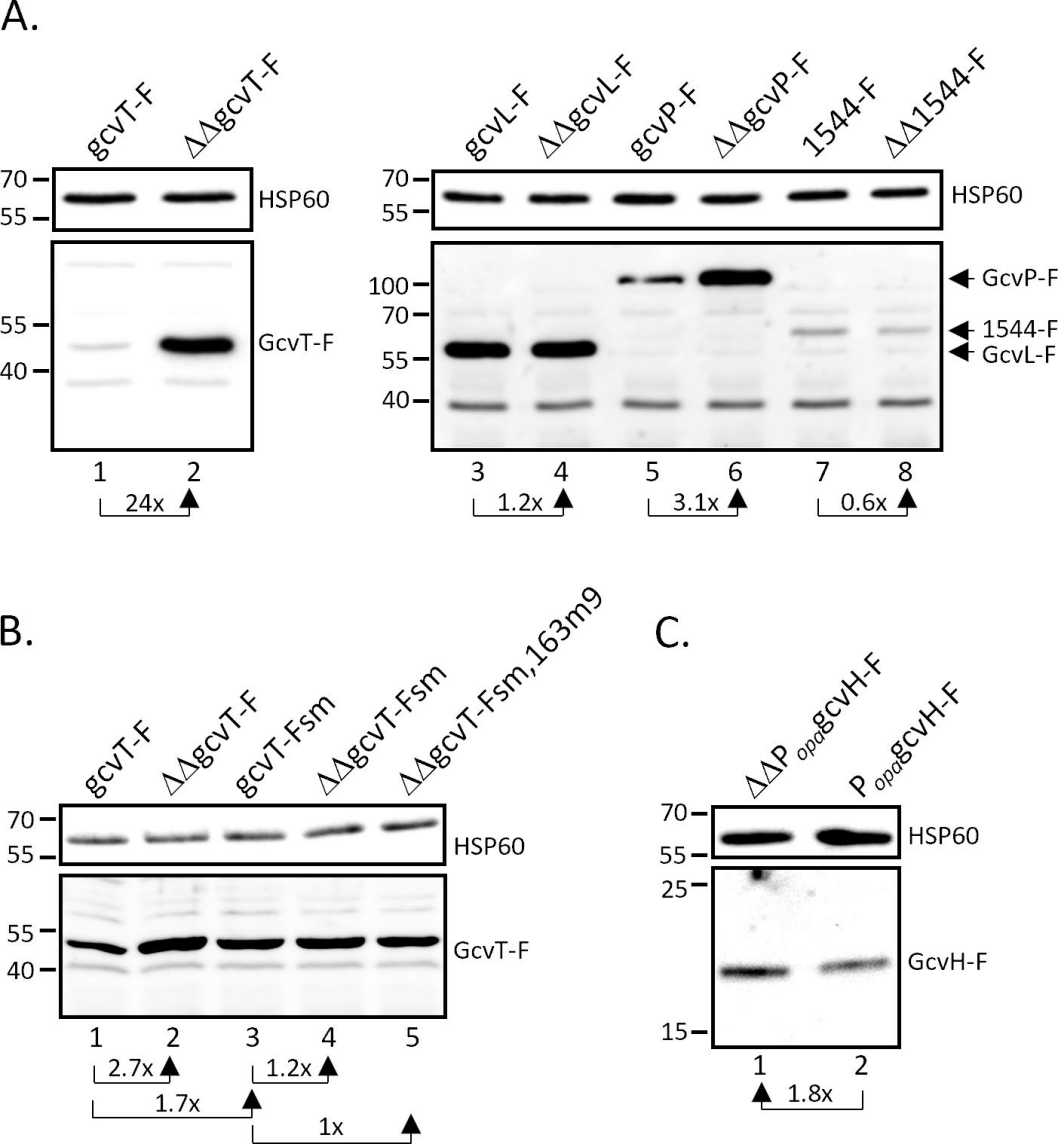
Expression of GCV system components in the presence and absence of the sibling sRNAs. (**A**) Expression of *gcvT*, *gcvP*, *gcvL,* and NGFG_01544. Derivatives of *N. gonorrhoeae* MS11 (lanes 1, 3, 5, and 7) and MS11 ΔΔ162/163 (lanes 2, 4, 6, and 8) expressing C-terminally 3xFLAG-tagged *gcvT* (lanes 1 and 2), *gcvL* (lanes 3 and 4), *gcvP* (lanes 5 and 6), or NGFG_01544 (lanes 7 and 8) were grown in PPM to an optical density of OD_550_ = 0.4 and protein lysates were prepared for Western blot analysis. (**B**) Impact of mutations affecting the predicted region of complementarity between *gcvT* mRNA and the sibling sRNAs. Strains MS11 gcvT-F, ΔΔgcvT-F, gcvT-Fsm, ΔΔgcvT-Fsm, and ΔgcvT-Fsm,163m9 were grown to OD_550_ = 0.4 in CDM10 containing 2.5 mM glycine, and protein lysates were prepared. (**C**) Expression of *gcvH* in MS11 P_opa_gcvH-F and ΔΔP_opa_gcvH-F. Protein lysates from *N. gonorrhoeae* cultures grown in PPM to OD_550_ = 0.4 were used for Western blotting. Immunoblotting was performed using a monoclonal antibody directed against the 3xFLAG epitope. Hybridization with a monoclonal antibody directed against HSP60 was performed as a loading control. Protein samples for the detection of 3xFLAG-tagged proteins and HSP60 were run on separate gels. Panels A, B, and C show the results from representative experiments (*n* = 3). The mean relative change in protein levels in the absence of the sibling sRNAs (panels **A**, **B, and C**) or as a consequence of alterations in the *gcvT* 5′-UTR and coding sequence or NgncR_163 sequence (panel B) is indicated. The position of the 3xFLAG-tagged target proteins is indicated on the right. Numbers on the left side of the panels indicate the position of size marker proteins (kDa).

To investigate post-transcriptional regulation by the sibling sRNAs, C-terminally 3xFLAG-tagged derivatives of these putative target genes were introduced into the *N. gonorrhoeae* wild-type strain MS11 and the sRNA double deletion mutant ΔΔ162/163. In strains MS11 gcvT-F and ΔΔgcvT-F, the *gcvT* downstream genes NGFG_01513 and *gcvH* were deleted; however, qRT-PCR analysis of *gcvT* mRNA levels in strain MS11 gcvT-F and mutant MS11 gcvH-F comprising the full-length *gcvT*-NGFG_01513-*gcvH* operon demonstrated that truncation of the operon did not affect *gcvT* transcription (Fig. S3). Consistent with the predicted blockade of the RBS, immunoblot analysis of bacteria cultured in PPM revealed considerable upregulation of GcvT and GcvP in the sRNA double deletion mutants, while the expression of GcvL remained unchanged. The amount of NGFG_01544 was slightly, but consistently, reduced in the absence of the sibling sRNAs (Fig. 1A). Post-transcriptional regulation of *gcvT* and *gcvP* was also investigated in *E. coli* using plasmid-encoded translational target *gfp* fusions (38). Co-expression of sRNA NgncR_162 resulted in slight downregulation of both GcvT-GFP and GcvP-GFP (Fig. S4). To validate the *in silico* predicted interaction between the sibling sRNAs and *gcvT* mRNA, strain MS11 gcvT-Fsm was constructed in which the region ranging from position −14 to +10 with respect to the translational start codon of *gcvT* was mutated (GGAGAAACTTGAGAATGACTGCTC to GGAGA**TCGC**TGAGAATGAC**ACTAG**; see Fig. S1A). In this mutant, *gcvT* expression was upregulated, indicating compromised sRNA binding (Fig. 1B). Consistently, deletion of the sibling sRNAs (strain MS11 ΔΔgcvT-Fsm) caused no further increase in GcvT. However, upon complementation of MS11 ΔΔgcvT-Fsm with a mutated derivative of NgncR_163 restoring complementarity between the sRNA and *gcvT* mRNA within the 5′-UTR (position −6 to −14; strain MS11 gcvT-Fsm,163m9), downregulation of GcvT was not observed (Fig. 1B), while complementarity in this region was sufficient for post-translational regulation of *gcvT* by sRNA NgncR_162 in *E. coli* (Fig. S1A and S4A). We therefore speculate that nucleotide exchanges might have compromised sRNA secondary structure and stability, though no such effects were predicted by RNAfold analysis (http://rna.tbi.univie.ac.at/cgi-bin/RNAWebSuite/RNAfold.cgi).

Since *gcvT* and *gcvH* are co-transcribed, the previously observed regulation of *gcvH* by the sibling sRNAs might not be caused by direct sRNA-mRNA interactions within the CDS as predicted by IntaRNA analysis (17), but might be rather due to an indirect effect on transcript stability caused by hybridization of the sibling sRNAs to the 5′-UTR of *gcvT*. To test the direct regulation of *gcvH*, the CDS of 3xFLAG-tagged *gcvH* was combined with an artificial upstream sequence consisting of position −80 to −15 of the *gcvT* 5′-UTR and the 13 base pairs located immediately upstream of the *gcvH* start codon. In the respective strains MS11 P_opa_gcvH-F and ΔΔP_opa_gcvH-F, transcription of *gcvH* is under the control of the promoter of a *Neisseria opa* gene. According to IntaRNA analysis, the sibling sRNAs will not hybridize to this artificial 5′-UTR, while predicted binding to the CDS is unaltered. As shown in Fig. 1C, the amount of GcvH in these mutants still increased in the absence of the sibling sRNAs, suggesting that their interaction with the *gcvH* CDS contributes to post-transcriptional regulation. Taken together, we have corroborated glycine cleavage as an important metabolic pathway under the control of the sibling sRNAs, which target the core components of the glycine cleavage system *gcvP*, *gcvT,* and *gcvH*.

### A tandem glycine riboswitch mediates glycine-responsive expression of *gcvT* and *gcvH*

**Fig 2:**
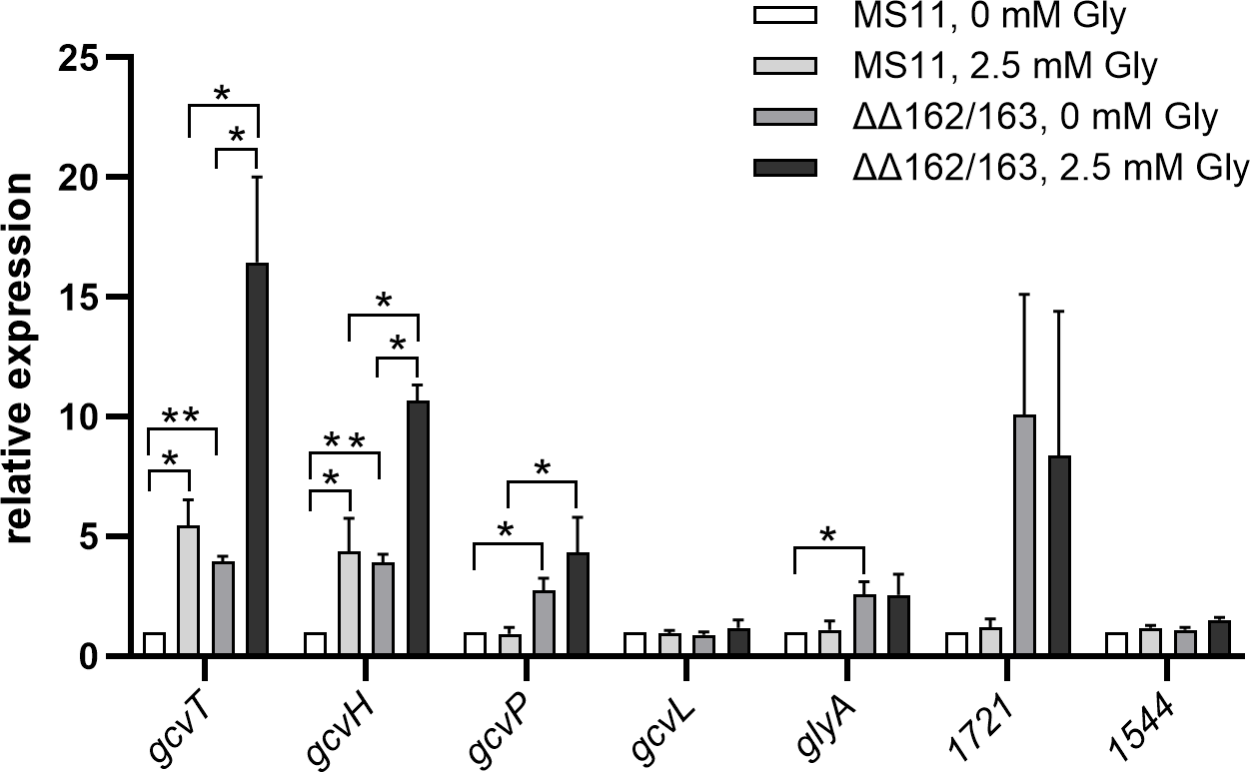
Analysis of glycine-responsive transcription of sibling sRNA targets. Transcript levels of *gcvT*, *gcvH*, *gcvP*, *gcvL*, *glyA*, NGFG_01721, and NGFG_01544 were analyzed by qRT-PCR in strains MS11 and MS11 ΔΔ162/163 cultivated in CDM10 medium containing either 0 mM or 2.5 mM glycine. The ratios (fold-change) of the transcript amount relative to the wild-type MS11 grown in the absence of glycine (normalized to a relative expression level of 1) are depicted. The indicated ratios represent the mean of the results of qRT-PCR experiments performed in triplicate on cDNAs obtained from three independent RNA preparations. Error bars indicate the standard deviation. Statistical significance was determined using Student’s t-test analysis (*=*P* < 0.05; **=*P* < 0.01; ***=*P* < 0.001).

**Fig 3:**
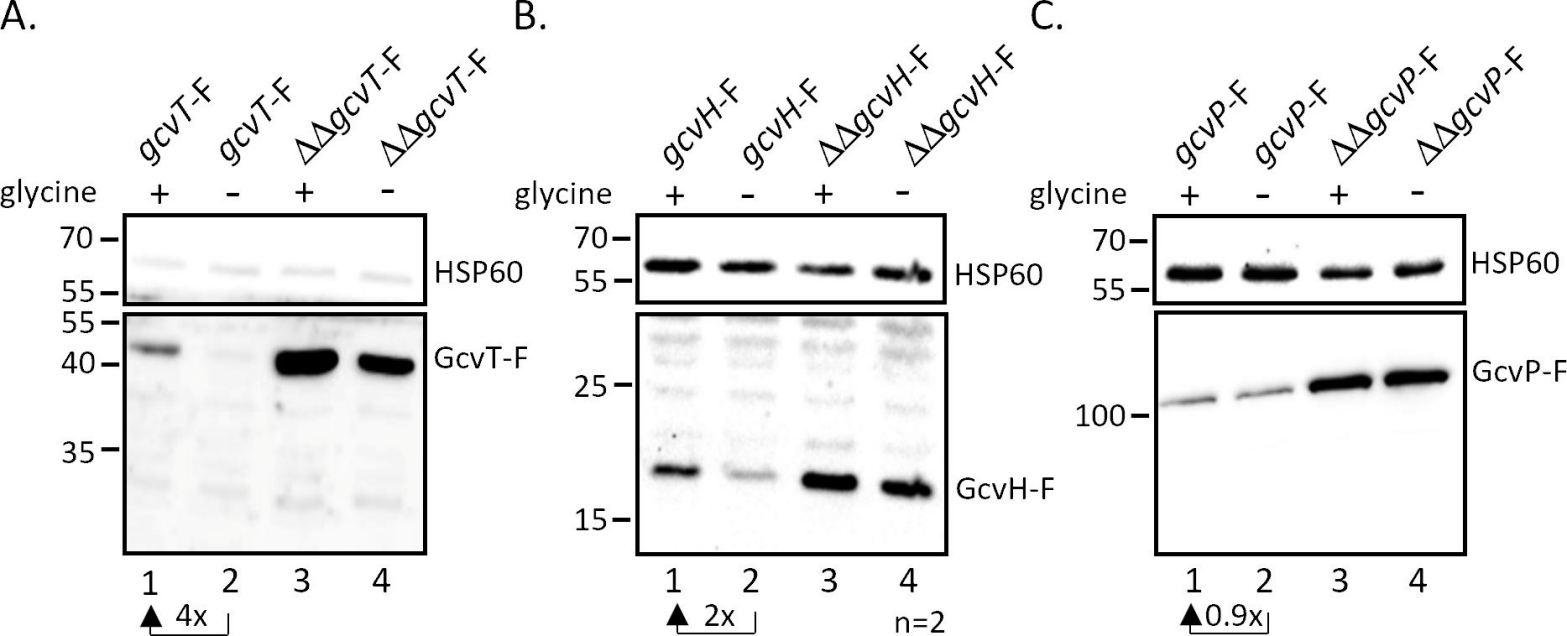
Expression of 3xFLAG-tagged GcvT, GcvH, and GcvP proteins in the presence and absence of glycine. Derivatives of MS11 wild-type (lanes 1 and 2) and MS11 ΔΔ162/163 (lanes 3 and 4) expressing C-terminally 3xFLAG-tagged *gcvT* (**A**), *gcvH* (**B**), or *gcvP* (**C**) were grown to OD_550_ = 0.4 in CDM10 medium containing 2.5 mM glycine (lanes 1 and 3) or 0 mM glycine (lanes 2 and 4). Bacteria were lysed, and Western Blot analysis was performed on equal amounts of protein using monoclonal antibodies directed against the 3xFLAG epitope, and HSP60 was used as a loading control. The results from representative experiments are shown (**A**,** C**, *n* = 3; B, *n* = 2), and the mean relative change in protein level in the presence of glycine in comparison to glycine-free medium is indicated for strains expressing 3xFLAG-tagged targets in the wild-type background. Numbers on the left side of the panel indicate the position of size marker proteins (kDa). Protein samples for the detection of 3xFLAG-tagged proteins and HSP60 were run on separate gels in **A** and** C**.

To investigate the physiological relevance of the above data, we tested whether glycine availability affects the expression of genes involved in its uptake and metabolism. Growth experiments demonstrated similar proliferation of gonococci in standard CDM10 medium (0.3 mM glycine), CDM10 medium lacking glycine or containing glycine in a concentration of 2.5 mM (Fig. S5). MS11 wild-type and mutant ΔΔ162/163 were cultivated in modified CDM10 medium either lacking glycine or containing glycine at a concentration of 2.5 mM, and qRT-PCR experiments were performed on RNA extracted from the bacteria. *gcvT* and *gcvH* mRNA levels increased in the presence of glycine in both wild-type and sRNA double deletion mutant, while no glycine-dependent expression changes were noted in the case of *gcvL*, *glyA*, NGFG_01721, and NGFG_01544 (Fig. 2). *gcvP* mRNA was not affected by glycine in MS11; however, we repeatedly noted slight upregulation in response to glycine in the absence of the sibling sRNAs. Glycine-dependent expression of *gcvT* and *gcvH* was confirmed by immunoblot analysis of protein lysates from strains encoding 3xFLAG-tagged derivatives of the respective protein, while glycine had no effect on GcvP protein level in both wild-type and sRNA double deletion mutant (Fig. 3).

**Fig 4:**
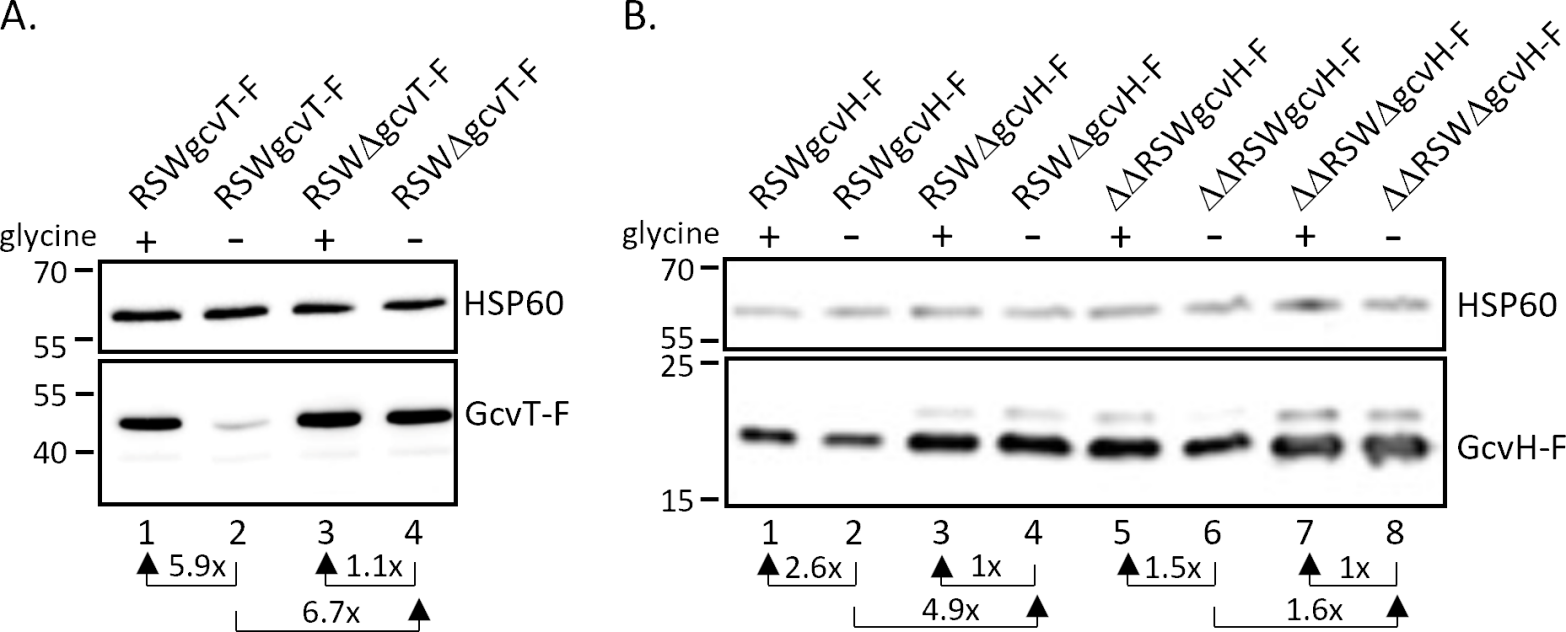
Glycine-responsive expression of 3xFLAG-tagged *gcvT* or *gcvH* under control of P*_opa_* in *N. gonorrhoeae* mutants with full-length (RSW) or truncated (RSWΔ) *gcvT* 5′-UTR. (**A**) Expression of *gcvT-*3F in the presence of the sibling sRNAs. Mutants RSWgcvT-F (lanes 1 and 2) and RSWΔgcvT-F (lanes 3 and 4) were cultivated in CDM10 medium in the presence (2.5 mM; lanes 1 and 3) or absence of glycine (lanes 2 and 4) to OD_550_ = 0.4. Equal amounts of protein from lysed bacteria were separated on 10% SDS polyacrylamide gels. Protein samples for the detection of GcvT-F and HSP60 were run on separate gels. (**B**) Expression of *gcvH*-3F in derivatives of wild-type MS11 and the sRNA double deletion mutant ΔΔ162/163. Equal amounts of proteins from mutants RSWgcvH-F (lanes 1 and 2), RSWΔgcvH-F (lanes 3 and 4), ΔΔRSWgcvH-F (lanes 5 and 6), and ΔΔRSWΔgcvH-F (lanes 7 and 8) cultivated in CDM10 medium with 2.5 mM glycine (lanes 1, 3, 5, and 7) or 0 mM glycine (lanes 2, 4, 6, and 8) were separated on a 12% SDS polyacrylamide gel. Immunoblotting was performed with monoclonal antibodies directed against the 3xFLAG epitope or HSP60 used as a loading control. The results from representative experiments are shown (*n* = 3), and the mean relative change in protein level in the presence of glycine in comparison to glycine-free medium is indicated. The relative expression change due to the truncation of the *gcvT* 5′-UTR is also shown. Numbers on the left side of the panel indicate the position of size marker proteins (kDa).

Based on bioinformatic analysis, a glycine-binding aptamer (aptamer 1) ranging from positions −308 to −211 (relative to the *gcvT* start codon) had been predicted in the 5′-UTR of *gcvT* by Remmele et al. (8). In the same study, the transcriptional start site of *gcvT* was mapped to position −302. Rfam analysis (https://rfam.org/) confirmed the prediction of a glycine riboswitch in the indicated region (e-value = 1.6e-12). Closer inspection of the 5′-UTR revealed the presence of a second putative glycine-binding aptamer sequence (aptamer 2) immediately downstream and ranging from position −213 to −55, which, however, shows less similarity to the glycine riboswitch consensus (Rfam e-value = 1.6e-6). Similar sequence motifs were also detected in the *gcvT* 5′-UTR of other members of the genus *Neisseria* (Fig. S6). To test whether *gcvT* and *gcvH* are indeed under control of a tandem glycine riboswitch, we constructed derivatives of strain MS11 with full-length or truncated *gcvT* 5′-UTR (encompassing position −80 to −1) expressing either 3xFLAG-tagged *gcvT* (with concomitant deletion of NGFG_01513 and *gcvH*) or *gcvH*. To rule out the effects of glycine-dependent regulation of transcription initiation, in these constructs, the *gcvT* promoter was replaced by the promoter of a *Neisseria opa* gene (indicated by “RSW” in the strain designation). As described above for strains expressing *gcvT* and *gcvH* under control of the P*_gcvT_* promoter (Fig. 3), higher amounts of the respective protein were detected by Western Blot analysis in strains RSWgcvT-F and RSWgcvH-F, when gonococci were grown in the presence of 2.5 mM glycine compared to cultivation without glycine (Fig. 4). In strains RSWΔgcvT-F and RSWΔgcvH-F with truncated *gcvT* 5′-UTR expression of *gcvT* and *gcvH* was no longer responsive to glycine availability and increased about sixfold and fivefold compared to control strains RSWgcvT-F and RSWgcvH-F grown in the absence of glycine. In the case of *gcvH,* the amount of 3xFLAG-tagged protein expressed in the strain with truncated *gcvT* 5′-UTR also exceeded that of the control strain grown in the presence of glycine, suggesting that a sequence motif being part of an inhibitory secondary structure formed in the 5′-UTR has been removed by the deletion. As expected, knockout of the sibling sRNAs in strains ΔΔRSWgcvH-F and ΔΔRSWΔgcvH-F resulted in a further increase in protein expression (Fig. 4B). Thus, we concluded that in addition to control by the sibling sRNAs, the *gcvT*-NGFG_01513-*gcvH* operon is indeed under control of a riboswitch functioning as an ON-switch to restrict the expression in the absence of glycine. To prove that the glycine riboswitch is solely responsible for glycine-responsive expression of *gcvT* and *gcvH,* we constructed a derivative of strain MS11 gcvT-F with deletion of positions −19 to −242 of the *gcvT* 5′-UTR sequence (corresponding to aptamer 2 and part of aptamer 1). In this construct, (MS11 P*_gcvT_*ΔgcvT-F), a segment of 76 base pairs located immediately downstream of the *gcvT* promoter −10 box, is retained to enable putative transcriptional regulator binding. When *gcvT* expression in the absence and presence of glycine was monitored in mutant P*_gcvT_*ΔgcvT-F, no changes in the amount of GcvT protein could be detected (Fig. S7).

### Characterization of the *N. gonorrhoeae gcvT* tandem glycine riboswitch

**Fig 5:**
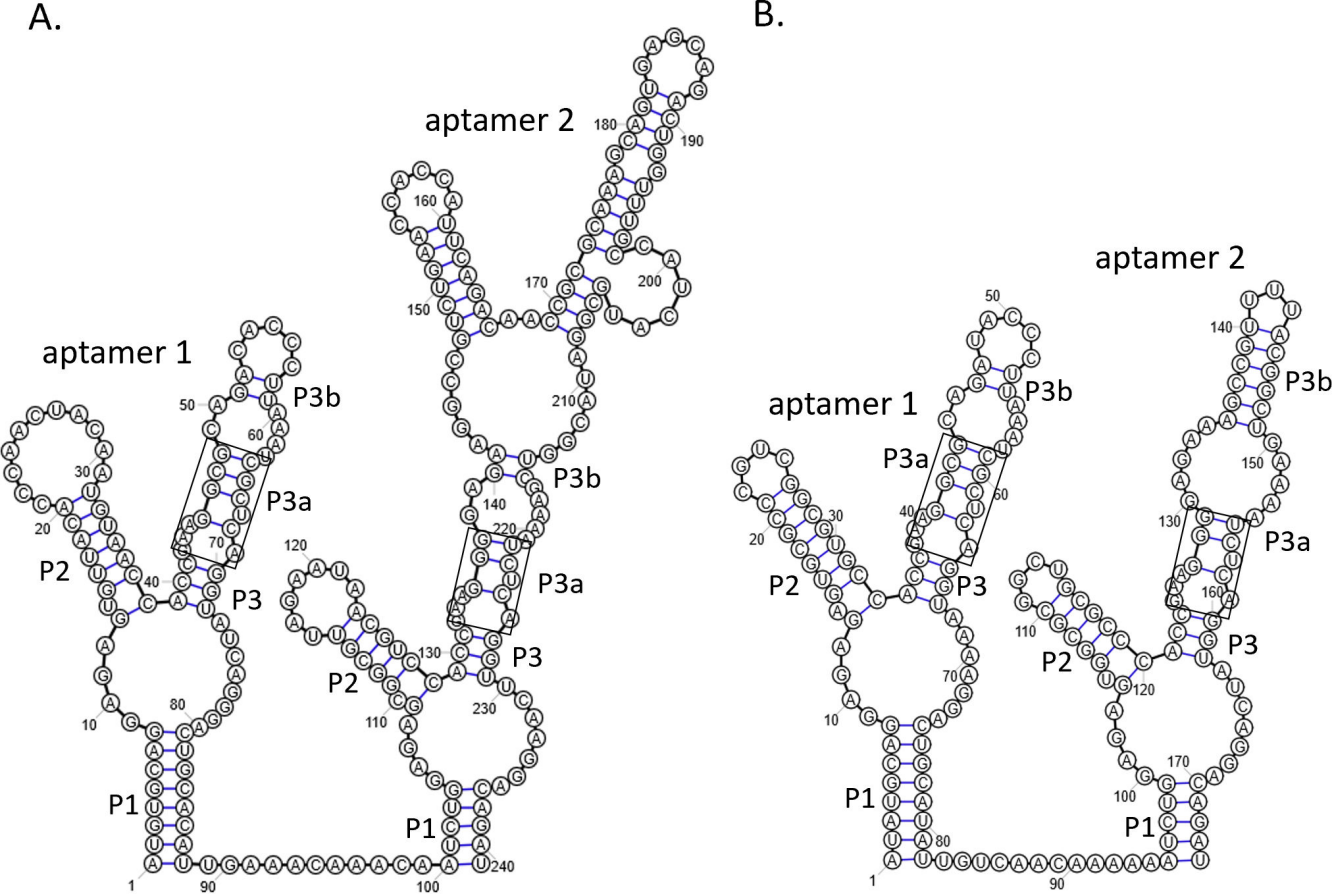
Predicted secondary structure of the *gcvT* tandem glycine riboswitch of *N. gonorrhoeae* (**A**) and *N. arctica* (**B**). Secondary structures were predicted using Rfam (https://rfam.org/) and modified according to current consensus structures (29, 42). Structure prediction of the extended P3 stem-loop of *N. gonorrhoeae* aptamer 2 was performed using RNAfold (http://rna.tbi.univie.ac.at/cgi-bin/RNAWebSuite/RNAfold.cgi). The P1, P2, and P3 helices are indicated, and glycine-binding sites are boxed. Secondary structures were drawn using RiboSketch (43).

**Fig 6:**
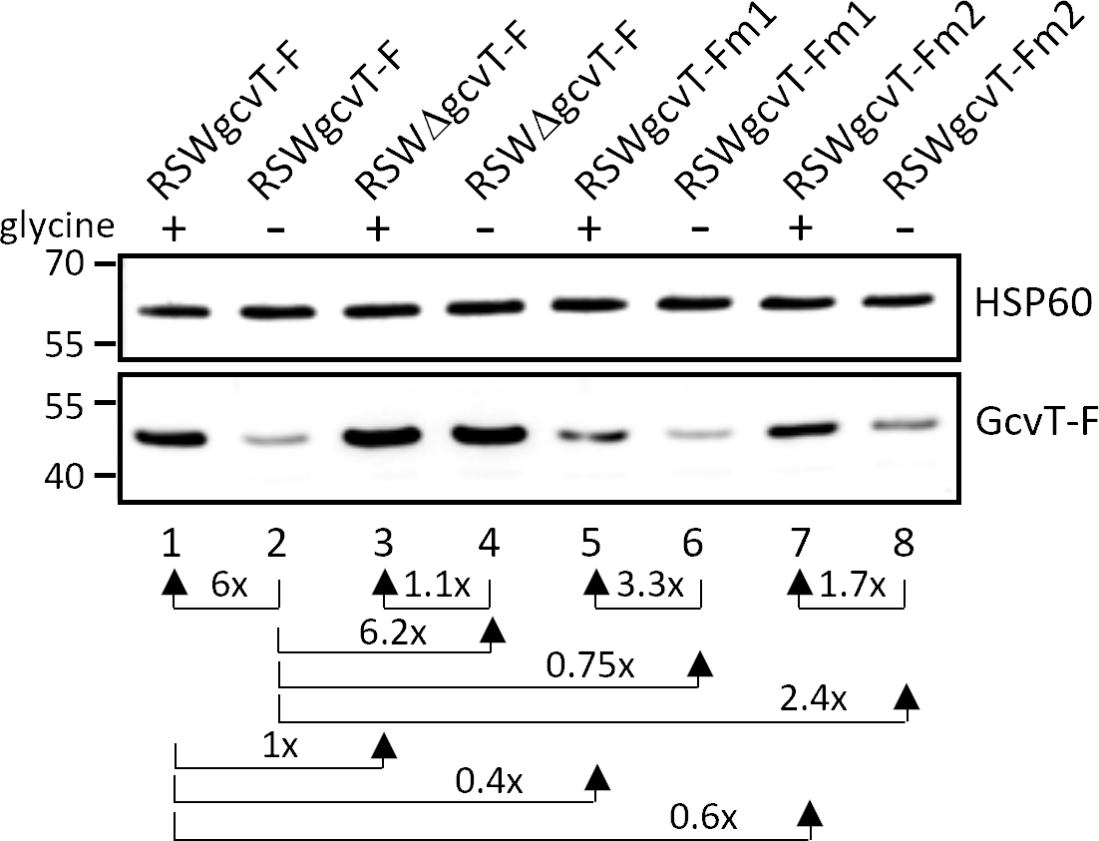
Glycine-responsive expression of 3xFLAG-tagged *gcvT* in *N. gonorrhoeae* strains with mutations in the glycine-binding pocket of aptamer 1 and aptamer 2. Mutants MS11 RSWgcvT-Fm1 (lanes 5 and 6) and RSWgcvT-Fm2 (lanes 7 and 8) carrying T to G substitutions at positions 67 (m1) and 224 (M2) of the glycine riboswitch sequence were cultivated in CDM10 medium in the presence (2.5 mM; lanes 5 and 7) or absence of glycine (lanes 6 and 8) to OD_550_ = 0.4. Strains RSWgcvT-F (lanes 1 and 2) and RSWΔgcvT-F (lanes 3 and 4), used as controls, were treated equally. Equal amounts of protein from lysed bacteria were separated on 10% SDS polyacrylamide gels. Protein samples for the detection of GcvT-F and HSP60 by immunoblotting were run on separate gels. The results from a representative experiment are shown (*n* = 3). Relative expression changes between strains and conditions (indicated by arrows) represent the mean of three independent experiments. Numbers on the left side of the panel indicate the position of size marker proteins (kDa).

Aminomethyltransferases of GCV systems are frequently controlled by tandem glycine riboswitches with two ligand-binding aptamers followed by a single expression platform (29). Each aptamer of a tandem glycine riboswitch adopts a secondary structure consisting of three helical segments denoted P1, P2, and P3, which converge around a central loop. Helix P3 contains the glycine-binding pocket, which is formed by an asymmetric A-rich bulge at the base of the P3a helix. Helix P3a comprises four base pairs, three of which are conserved as canonical GC pairs, while the fourth is typically a purine-U pair. A bulged uracil located in the P3a helical segment is also crucial for glycine binding (39). The two aptamers form a semisymmetric dimer via long-range tertiary interactions, including A-minor motifs which occur between adenines in the P3 helix of either aptamer and the P1 helix of the adjacent aptamer, and a semiconserved Hoogsteen base pair between nucleotides in the P3a/3b junction of each aptamer (39). While aptamer 1 of the *N. gonorrhoeae* tandem glycine riboswitch corresponds well to the consensus, some divergence is apparent in aptamer 2: the P3a helix is shortened to three base pairs, only two of which are GC pairs, and, most notably, the “P3 loop sequence” comprises 73 nucleotides which according to RNAfold analysis might adopt a Y-like shape by forming two stem-loop structures (Fig. 5A). Interestingly, comparison of the riboswitch sequences in the *gcvT* 5′-UTR of other members of the genus *Neisseria* revealed some variability in this region of aptamer 2 with the sequence of *N. arctica* corresponding best to the consensus (Fig. S6; Fig. 5B). The kink-turn and P0-helix, which are frequently part of tandem glycine riboswitches (20, 40) and formed by sequence motifs located at the 5′-end of the riboswitch and immediately downstream of aptamer 1, are apparently not present in *N. gonorrhoeae*. However, a pseudoknot formed between a polycytidine tract in the P3b stem-loop and a polyguanine tract immediately following aptamer 2, which was found in 66% of aminomethyltransferase glycine riboswitches analyzed by Torgerson et al. (29), might also be present in *N. gonorrhoeae* (Fig. S6). A single binding event in either aptamer of the aminomethyltransferase tandem glycine riboswitch of *Bacillus subtilis* was reported to be sufficient to promote helical switching; however, aptamer 1 binding was required for robust downstream gene expression *in vivo* (29, 41). Since it was shown that uracil residues 81 and 197 in the glycine-binding pockets of the *B. subtilis gcvT* tandem riboswitch are crucial for glycine binding (29), we mutated the corresponding uracil residues in the glycine-binding pocket of aptamer 1 (U67) or aptamer 2 (U224) to guanine in strain RSWgcvT-F, creating mutants RSWgcvT-Fm1 and RSWgcvT-Fm2, and analyzed glycine responsive expression of 3xFLAG-tagged GcvT by Western blotting (Fig. 6). Aptamer 1 mutant RSWgcvT-Fm1 still responded to glycine; however, GcvT expression was reduced to about 40% of the amount detected in the presence of 2.5 mM glycine in strain RSWgcvT-F comprising the wild-type riboswitch sequence. Mutation of the glycine-binding pocket of aptamer 2 resulted in a twofold increase in GcvT expression in the absence of glycine, suggesting that this mutation might affect the structure of the expression platform. GcvT level in the presence of glycine was closer to the wild-type in the aptamer 2 than in the aptamer 1 mutant.

**Fig 7:**
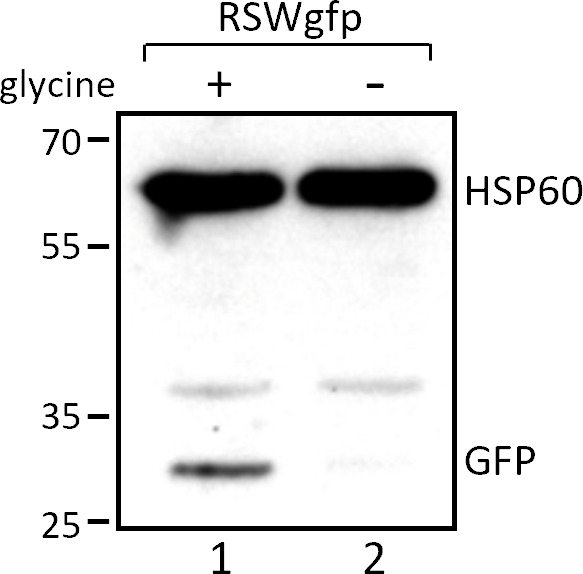
Analysis of *gfp* expression in MS11 RSWgfp harboring a fusion of *gfp* to the 5′-UTR of *gcvT*. Gonococci were grown in CDM10 medium containing 2.5 mM (lane 1) or no glycine (lane 2) to an OD_550_ = 0.4. Bacteria were lysed, and equal amounts of proteins were analyzed by Western blot using monoclonal antibodies directed against GFP and HSP60 used as a loading control. The figure shows the result from a representative experiment (*n* = 2). Numbers on the left side of the panel indicate the position of size marker proteins (kDa).

Structural changes in the expression platform of the riboswitch induced by ligand binding to the aptamer(s) may affect transcription or translation attenuation or even dual transcription and translation attenuation (44). No Rho-independent transcription terminator hairpin is predicted in the *gcvT* mRNA downstream of the riboswitch. To test whether ligand binding might affect Rho-dependent transcription termination, which has been first reported for the Mg^2+^- and FMN-binding riboswitches of *Salmonella enterica* serovar Typhimurium and *E. coli* (34), we cultivated *N. gonorrhoeae* mutants MS11 RSWgcvT-F and RSWΔgcvT-F in the absence and presence of 10 mM bicyclomycin, which is an inhibitor of the termination factor Rho. Growth of gonococci was not affected by the drug, and expression of *gcvT* still responded to glycine availability in strain RSWgcvT-F comprising the full-length *gcvT* 5′-UTR (Fig. S8). Furthermore, RT-PCR experiments performed with amplicons derived from the 5′-UTR or the *gcvT* and *gcvH* CDS did not provide indications that glycine-responsive expression of the GCV system components is caused by premature transcription termination occurring in the absence of glycine (data not shown). Therefore, we concluded that ligand binding to the *gcvT* glycine riboswitch controls the initiation of translation. To test whether all structural elements required for the ON/OFF switch are confined within the *gcvT* 5′-UTR, we replaced *gcvT* (and the remainder of the operon) with *gfp*. In addition, in the respective mutant MS11 RSWgfp, the P*_gcvT_* promoter was replaced by P*_opa_*. As shown in Fig. 7, glycine-responsive expression of *gfp* was observed in strain MS11 RSWgfp, demonstrating that the *gcvT* CDS lacks regulatory elements.

**Fig 8:**
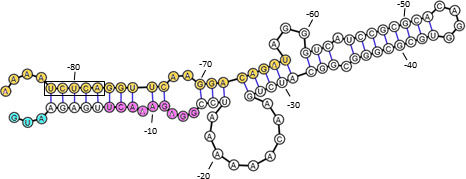
Secondary structure prediction of part of the *gcvT* 5′UTR comprising nucleotides at positions −86 to +3 suggests participation of the aptamer 2 glycine-binding pocket in the formation of the OFF-state of the riboswitch expression platform. Secondary structure prediction was performed using RNAfold. The *gcvT* start codon is highlighted in cyan, the region of complementarity to the sibling sRNAs is shown in magenta, and nucleotides from aptamer 2 of the glycine riboswitch are shown in yellow. Nucleotides from the glycine-binding pocket are boxed. RiboSketch (43) was used for secondary structure drawing.

**Fig 9:**
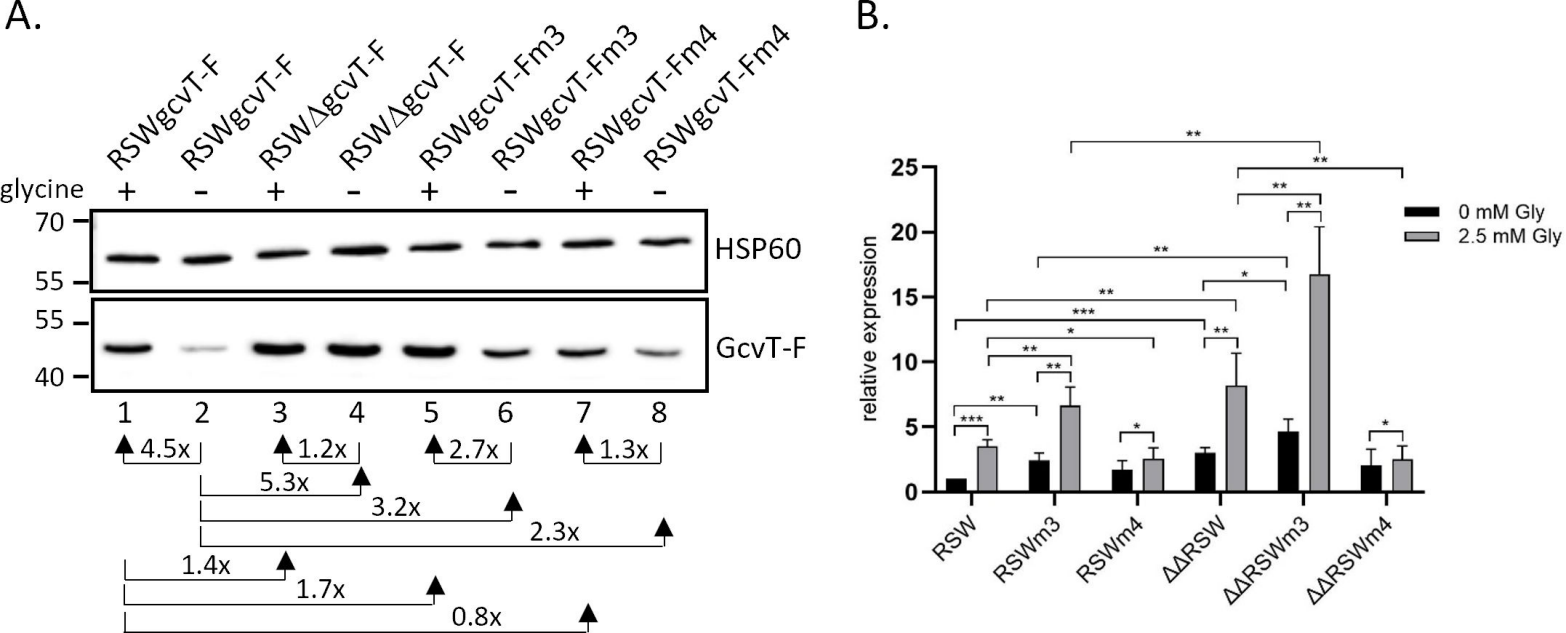
Glycine-responsive expression of *gcvT* in *N. gonorrhoeae* strains with mutations in the immediate upstream region of *gcvT*. (**A**) Immunoblot analysis of *gcvT* expression in *N. gonorrhoeae* strains harboring 3xFLAG-tagged *gcvT*. Control strains MS11 RSWgcvT-F (lanes 1 and 2) and RSWΔgcvT-F (lanes 3 and 4) and mutants RSWgcvT-Fm3 (lanes 5 and 6) and RSWgcvT-Fm4 (lanes 7 and 8) were cultivated in CDM10 medium in the presence (2.5 mM; lanes 1, 3, 5, and 7) or absence of glycine (lanes 2, 4, 6, and 8) to OD_550_ = 0.4. Equal amounts of protein from lysed bacteria were separated on 10% SDS polyacrylamide gels. Protein samples for the detection of GcvT-F and HSP60 by immunoblotting were run on separate gels. The results from a representative experiment are shown (*n* = 3). Relative expression changes between strains and conditions (indicated by arrows) represent the mean of three independent experiments. Numbers on the left side of the panel indicate the position of size marker proteins (kDa). (**B**) Quantification of *gcvT* mRNA transcribed from *gcvT* alleles with mutated 5′-UTR in the presence or absence of the sibling sRNAs by qRT-PCR. Control strains MS11 RSW and ΔΔRSW and *gcvT* 5′-UTR mutants MS11 RSWm3, RSWm4, ΔΔRSWm3, and ΔΔRSWm4 were cultivated in CDM10 medium containing either 0 mM or 2.5 mM glycine to an OD_550_ = 0.4, and RNA was extracted for qRT-PCR analysis. The ratios (fold-change) of the transcript amount relative to the control strain MS11 RSW grown in the absence of glycine (normalized to a relative expression level of 1) are depicted. The indicated ratios represent the mean of the results of qRT-PCR experiments performed in triplicate on cDNAs obtained from five independent RNA preparations. Error bars indicate the standard deviation. Statistical significance was determined using Student’s t-test analysis (*=*P* < 0.05; **=*P* < 0.01; ***=*P* < 0.001).

Secondary structure prediction of the *gcvT* 5′-UTR (nucleotides −86 to +3) revealed complementarity with the capacity to form an extended hairpin (Fig. 8). Part of the hairpin is formed between a segment of aptamer 2, including nucleotides from the glycine-binding pocket and the immediate upstream sequence of *gcvT* (positions −1 to −11). Since both in the aptamer 2 mutant and strain RSWΔgcvT-F, transcribing an mRNA lacking the respective aptamer 2 segment GcvT was upregulated in the absence of glycine, we hypothesized that this region of complementarity might be part of the expression platform. Surprisingly, this region, possibly involved in glycine-responsive regulation, overlaps with the sequence motif predicted to be targeted by the sibling sRNAs (positions −6 to −14). Therefore, strains RSWgcvT-Fm3 and RSWgcvT-Fm4 with mutagenized sequence motifs corresponding to positions −5 to −2 (m3, TGAG to GACA) and −10 to −1 (m4, AAACTTGAGA to CTCACCACAT; nucleotides predicted to be involved in sRNA binding are underlined) of the *gcvT* 5′-UTR were created to compromise formation of a putatively inhibitory secondary structure. In fact, immunoblot analysis revealed the upregulation of GcvT in the absence of glycine in both mutants (m3: 3-fold, m4: 2-fold), which, however, was less pronounced than in strain RSWΔgcvT-F with the truncated riboswitch (Fig. 9A). While strain RSWgcvT-Fm3 still responded to glycine and even showed somewhat higher GcvT expression than the control strain with wild-type *gcvT* 5′-UTR, glycine responsiveness was largely abolished in strain RSWgcvT-Fm4. Since post-transcriptional regulation by both riboswitch and sRNAs is supposed to be affected in mutant RSWgcvT-Fm4, we expected an additive effect resulting in more pronounced deregulation of target gene expression. However, relief of negative control due to eliminated complementarity might be counteracted by compromised ribosome binding due to the m4 mutation close to or overlapping the RBS. Furthermore, *gcvT* mRNA levels were analyzed by qRT-PCR in strains MS11 RSWm3, ΔΔRSWm3, RSWm4, and ΔΔRSWm4, which express *gcvT* with m3 or m4 5′-UTR mutation under control of P*_opa_* in the presence (RSW) or absence (ΔΔRSW) of the sibling sRNAs. As expected, deletion of the sibling sRNAs resulted in the upregulation of *gcvT* in both the RSW control strain with wild-type *gcvT* 5′-UTR and MS11 RSWm3, in which the predicted sRNA-binding site is unaffected by the mutation. In contrast, *gcvT* mRNA levels were similar in mutants MS11 RSWm4 and ΔΔRSWm4, indicating that the m4 mutation overlapping the predicted sRNA interaction site indeed compromised sRNA-mediated post-transcriptional regulation (Fig. 9B). However, it should be noted that *gcvT* mRNA was less abundant in mutant ΔΔRSWm4 compared to strain ΔΔRSW, suggesting compromised translational initiation due to the m4 mutation.

**Fig 10:**
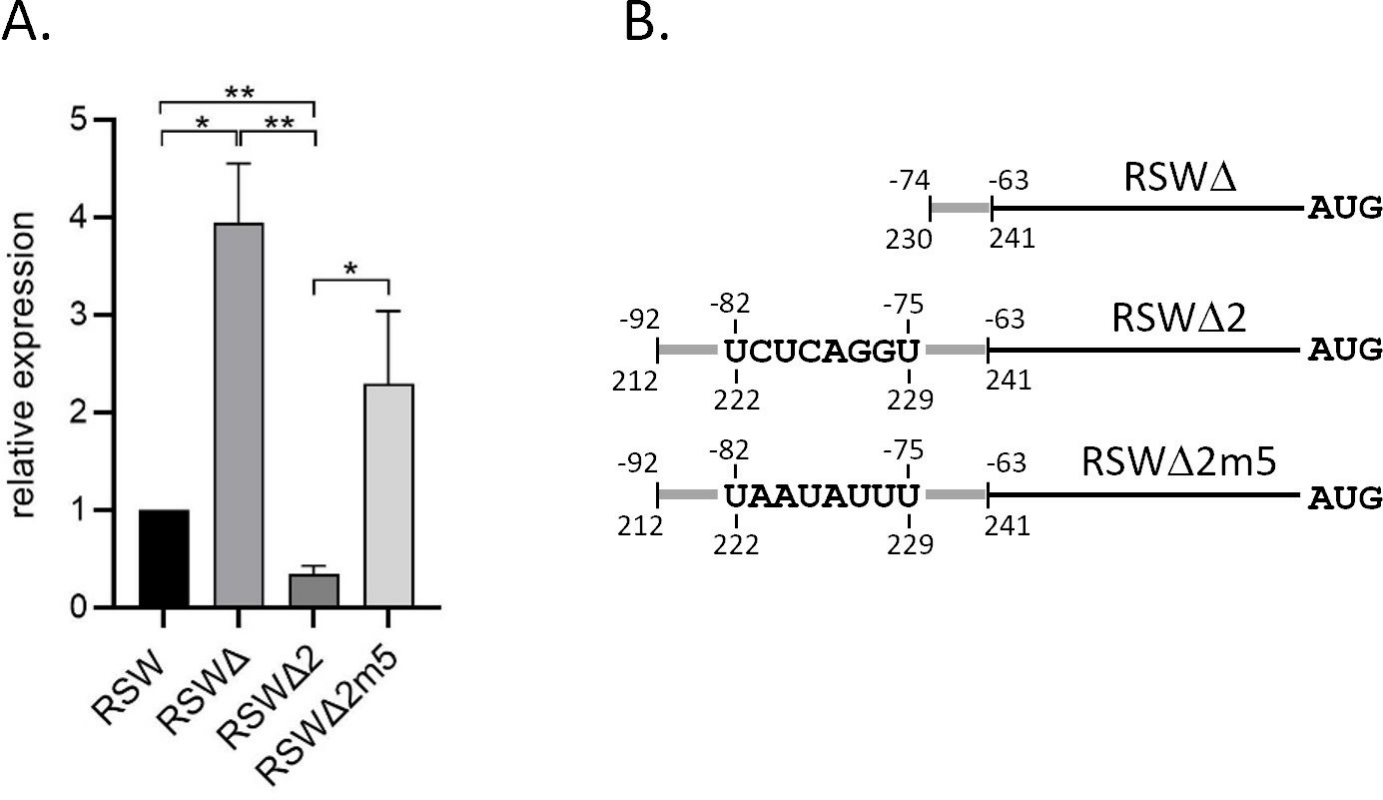
Analysis of *gcvT* transcript levels in *N. gonorrhoeae* mutants with truncated *gcvT* 5′-UTR. (**A**) Transcript levels of *gcvT* were analyzed by qRT-PCR in strains MS11 RSW, RSWΔ, RSWΔ2, and RSWΔ2m5 cultivated in CDM10 lacking glycine. *gcvT* mRNA from strain RSWΔ lacks the region of complementarity to the translation initiation region, while this sequence segment is part of the *gcvT* mRNA in strain RSWΔ2. The m5 mutation in RSWΔ2 obstructs base pairing to the translation initiation region. The ratios (fold-change) of mRNA amount relative to MS11 RSW (normalized to 1) are depicted. The indicated ratios represent the mean of the results of qRT-PCR experiments performed in triplicate on cDNAs obtained from three independent RNA preparations. Error bars indicate the standard deviation. Statistical significance was determined using Student’s t-test analysis (*=*P* < 0.05; **=*P* < 0.01; ***=*P* < 0.001). (**B**) Schematic representation of the 5′-UTR of the *gcvT* mRNA in mutants RSWΔ, RSWΔ2, and RSWΔ2m5. Thick gray lines indicate riboswitch sequence. Numbers above and below the lines indicate the distance to the *gcvT* start codon and nucleotide positions within the gonococcal tandem glycine riboswitch, respectively.

To confirm that the expression platform of the gonococcal glycine tandem riboswitch engages part of the aptamer 2 sequence in an inhibitory secondary structure, mutant MS11 RSWΔ2 was constructed in which a *gcvT*-NGFG_01513-*gcvH* mRNA with a 5′-UTR of 92 nucleotides capable of forming the predicted hairpin is transcribed. In contrast to mutant MS11 RSWΔ, in which the translation initiation region of the *gcvT* mRNA cannot be obstructed by base pairing, transcription of *gcvT* in the absence of glycine was not upregulated compared to the control strain MS11 RSW comprising the complete *gcvT* 5′-UTR (Fig. 10). However, when a mutation was introduced into the aptamer 2 sequence (CTCAGG to AATATT; positions −81 to −76 of the *gcvT* 5′-UTR), elevated amounts of *gcvT* mRNA could be detected in the respective mutant MS11 RSWΔ2m5 (Fig. 10), indicating relief of translational inhibition.

## DISCUSSION

In a recent study, we identified serine/glycine metabolism as a major pathway targeted by the *N. gonorrhoeae* sibling sRNAs (17). This finding is corroborated here by the observation that not only *gcvH*, but also other components of the GCV system, that is, *gcvT* and *gcvP,* are under post-transcriptional control of NgncR_162 and NgncR_163 (Fig. 1A). Upregulation of *gcvT* in a *hfq* deletion mutant of *N. meningitidis* has been reported previously (45, 46), which can be explained by strongly reduced sRNA levels in the absence of the RNA chaperone (47). Furthermore, not only the sibling sRNAs but also *gcvT* mRNA were demonstrated to bind Hfq in a RIP-seq analysis (47). From the data presented here, we conclude that binding of the sibling sRNAs to the *gcvH* coding region has only a minor effect (Fig. 1C) on post-transcriptional regulation, which seems to be mostly accomplished indirectly via sRNA binding to the *gcvT* 5′-UTR, which presumably affects the stability of the tricistronic mRNA. Although a region of complementarity between the coding sequence of *gcvL* and the sibling sRNAs is predicted *in silico* (Fig. S1C), we did not observe any effect of sRNA deletion on *gcvL* expression on mRNA or protein level (Fig. 1A and 2). This lack of regulation might be explained by the fact that in bacteria, dihydrolipoamide dehydrogenase GcvL, besides its role in glycine cleavage, is also involved in the 2-oxoacid dehydrogenase reaction (48). NGFG_01544 is annotated as M61 family aminopeptidase, members of which were reported to exhibit a preference for the cleavage of N-terminal glycine and alanine residues (49). Therefore, NGFG_01544 might contribute to satisfying the glycine demand of gonococci when free glycine is not available. While downregulation of NGFG_01544 in the sRNA double deletion mutant, which we observed in our recent transcriptome analysis (17), could not be confirmed by qRT-PCR (Fig. 2), a slight reduction in the amount of NGFG_01544 protein was detected in the absence of the sibling sRNAs (Fig. 1A). However, it seemed counterintuitive that NGFG_01544 expression is positively affected by the sibling sRNAs, while genes involved in glycine uptake and metabolism are negatively regulated (17). According to phylogenetic relationships, M61 family aminopeptidases can be classified into three branches. While branch 2 represents enzymes with a preference for N-terminal glycine and alanine residues, branch 1 comprises enzymes with homology to aminopeptidase BcepAP from *Burkholderia cepacia*, which preferentially cleaves off acidic amino acids (50). Interestingly, NGFG_01544 belongs to branch 1 (50) and therefore might not be involved in glycine metabolism.

**Fig 11:**
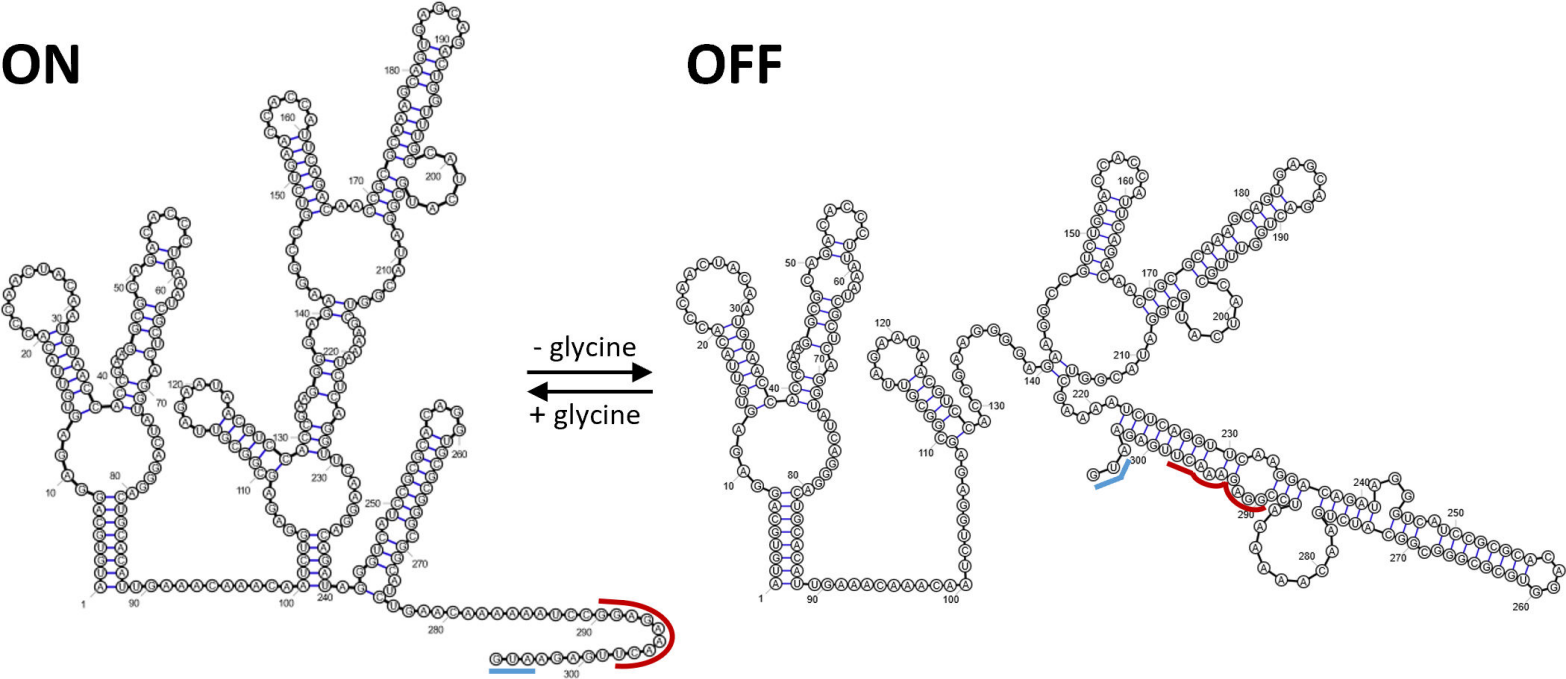
Model for ON- and OFF-switching of the *N. gonorrhoeae* translational *gcvT* tandem glycine riboswitch, based on mutational analysis. Glycine binding to aptamer 1 is supposed to assist in the folding of aptamer 2, which engages a stretch of nucleotides with complementarity to the translation initiation region of *gcvT*. In the absence of glycine, the translation initiation region is obstructed by base pairing to part of the riboswitch aptamer 2 sequence. The blue line indicates the position of the *gcvT* start codon. The region of complementarity to the sibling sRNAs NgncR_162/163 is marked by a red line. RiboSketch (43) was used for secondary structure drawing.

Neither expression of glycine transporter NGFG_01721 and other putative glycine transport proteins (NGFG_01699; NGFG_02166) nor of GlyA, which uses glycine for serine biosynthesis, was affected by the availability of glycine (Fig. 2 and data not shown). In contrast, expression of the GCV system proteins GcvT and GcvH is controlled via a glycine-responsive riboswitch, but surprisingly, GcvP, the glycine decarboxylase which acts in concert with GcvT and GcvH, did not show a robust glycine-responsive expression change. Riboswitch control of only a subset of *gcv* genes has also been observed in *B. subtilis* and *Listeria monocytogenes* (*gcvTPaPb* operon) (28, 31), while in *Burkholderia* spp. (*gcvTHP* operon) and *S. griseus* (both gcv*TH* operon and *gcvP*), regulation of all three components relies on this mechanism (26, 30). A recent bioinformatic analysis performed by Torgerson et al. (29) revealed that 81% of the tandem glycine riboswitches under study are located upstream of genes involved in glycine catabolism and are supposed to activate gene expression at high glycine concentrations, as demonstrated for the glycine riboswitches controlling GCV system genes in *B. subtilis*, *S. griseus,* and *Burkholderia* spp (26, 28, 30). Consistently, the *N. gonorrhoeae* translational tandem glycine riboswitch upstream of *gcvT-*NGFG_01513-*gcvH* functions as an ON-switch and exhibits features characteristic of this class. In fact, Torgerson et al. (29) reported notable differences in the structure of ON- and OFF-switch versions of the tandem glycine riboswitch, the latter typically controlling sodium-alanine symporters (28, 51): ON-switches exhibit a highly conserved glycine-binding pocket with three GC base pairs in aptamer 1 or both aptamers, while in OFF-versions, the glycine-binding pocket of aptamer 2 is highly conserved and that of aptamer 1 shows higher variability. Furthermore, the P5 pseudoknot is absent in the OFF-versions, but frequent in ON-riboswitches, whereas the kink-turn motif is more conserved in OFF-riboswitches. A high degree of conservation of one aptamer of a tandem glycine riboswitch depending on the regulated downstream gene was also observed by Crum et al. (42). In fact, singlet glycine riboswitches consisting of only one glycine-binding aptamer and a short hairpin termed “ghost aptamer,” which is located immediately upstream or downstream, can functionally be classified according to sequence similarity to the conserved aptamers 1 or 2 of tandem ON- and OFF-riboswitches (42). Interestingly, such singlet riboswitches exhibit glycine-binding affinities comparable to RNAs with tandem aptamer architecture (52). By mutational analysis combined with *in vitro* transcription termination assays, it was demonstrated that in the *B. subtilis gcvT* tandem glycine ON-riboswitch containing three and two GC base pairs in the binding pockets of aptamer 1 and 2, respectively, glycine binding to the first aptamer is favored and less dependent on tertiary interactions forming the aptamer dimer interface. Nevertheless, a single glycine-binding event in either aptamer was sufficient to promote helical switching linked to a change in gene expression in this experimental setting (29). Consistently, mutagenesis of the glycine-binding pocket of aptamer 1 of this riboswitch drastically reduced, but did not completely abolish glycine-responsive gene expression *in vivo*, while preventing glycine binding to aptamer 2 did not affect glycine-responsiveness or maximum gene expression (41). In the *N. gonorrhoeae* aptamer 1 mutant glycine still elicited an increase in the amount of GcvT protein to about one-third of the wild-type level, suggesting that aptamer 2 contributes substantially to helical switching despite its lower degree of conservation. In contrast to well-characterized transcriptional tandem glycine ON- and OFF-riboswitches (29, 39, 53), mutation of the aptamer 2 glycine-binding pocket of the *N. gonorrhoeae* riboswitch seems to directly affect the structure of the expression platform. Based on the following observations, it seems likely that in the OFF-state of the riboswitch system described here, nucleotides from the P3a, P3, and P1 stem base pair with a stretch of nucleotides located immediately upstream of the *gcvT* start codon to block translation initiation: (i) Mutagenesis of U224 in aptamer 2 or nucleotides at positions −2 to −5 of the full-length *gcvT* 5′-UTR enhanced *gcvT* expression in the absence of glycine (Fig. 6 and 9). (ii) Truncation of the *gcvT* 5′-UTR to an extent that retains the region of complementarity resulted in *gcvT* expression similar to wild-type level in the absence of glycine, while nucleotide exchanges corresponding to positions 223 to 229 of the aptamer 2 glycine-binding pocket, which compromise complementarity, increased *gcvT* expression (Fig. 10). Nevertheless, glycine binding to aptamer 1 still elicited an increase in *gcvT* expression in the aptamer 2 mutant, indicating structural rearrangements that lead to further stabilization of the ON-conformation of the expression platform. Similarly, glycine binding to the riboswitch induced further relief of translational hindrance in mutant RSWgcvT-Fm3. The nucleotide sequence of the proposed expression platform is not well conserved in all *Neisseria* species included in this study (Fig. S6). Three groups with similar *gcvT* 5′-UTRs can be distinguished, and, despite the sequence variability between the different groups, according to secondary structure predictions, in all cases, an extended stem-loop structure involving nucleotides from aptamer 2 can be formed. In *N. animalis* and *N. arctica,* the regions between the P1 helix of aptamer 2 and the *gcvT* start codon are considerably longer (116 and 185 nucleotides, respectively, versus 62 nucleotides in *N. gonorrhoeae*) and differ in sequence from all the other *Neisseria* species; however, base pairing between nucleotides from the aptamer 2 glycine-binding pocket and the immediate upstream region is predicted *in silico*. Taken together, we hypothesize that, as demonstrated for the glycine tandem ON-riboswitch of *B. subtilis* (29), high-affinity glycine binding to the first aptamer acts as a scaffold for the folding of aptamer 2, which ultimately establishes the ON-conformation of the *Neisseria* riboswitch (Fig. 11).

Surprisingly, base pairing interactions between the sibling sRNAs and the *gcvT* 5′-UTR occur in a region that seems to be part of the expression platform. Although, at least in the case of sRNA NgncR_163, more extended base pairing capability involving also the *gcvT* CDS is predicted, our data indicated that complementarity to nucleotides at positions −6 to −14 (-8 to −14 in case of NgncR_162) of the *gcvT* 5′-UTR is sufficient to confer post-transcriptional regulation (Fig. S4). This suggests considerable flexibility and dynamics in the secondary structure of the *gcvT* 5′-UTR, since the effect of sibling sRNA deletion is observed even in the absence of glycine when the immediate upstream sequence of *gcvT* is engaged in establishing the riboswitch OFF-state conformation (Fig. 2 to 4B and 9B). Unexpectedly, the RSWgcvT-Fm4 mutation, which should both compromise the OFF-structure of the riboswitch and abolish sRNA binding, did not raise *gcvT* expression to a level comparable to that observed in the sRNA double deletion mutant. However, this result may be a consequence of an impaired translation initiation caused by nucleotide exchanges within the RBS. Two-tier post-transcriptional control of the *gcvT*-NGFG_01513-*gcvH* operon allows for the integration of different signals to fine-tune expression of the GCV system in response to the metabolic needs of gonococci, that is, (i) glycine availability controlling the riboswitch and (ii) an yet unidentified stimulus governing transcription of the sibling sRNAs. These post-transcriptional control mechanisms might even act on top of transcriptional control exerted by a transcription factor. Interestingly, an Lrp/AsnC family transcriptional regulator is encoded immediately upstream of the *gcvT*-NGFG_01513-*gcvH* operon; however, deletion of the respective gene (NGFG_01511) did not affect *gcvT* and *gcvH* mRNA levels when gonococci were grown in PPM-rich medium (data not shown). From the observation that inactivation of the riboswitch is sufficient to abolish glycine-responsiveness of *gcvT* expression, we conclude that at least glycine is not a stimulus, which affects transcription initiation at the *gcvT* promoter (Fig. S7).

So far, reports on mRNAs regulated by both a riboswitch and an sRNA are scarce. The first example of an mRNA controlled by both cis- and trans-acting RNA-based regulatory elements was recently provided by Bastet et al. (54). In *E. coli,* an adenosylcobalamin (AdoCbl)-responsive OFF-riboswitch inhibits the translation of the *btuB* mRNA encoding an outer membrane corroid receptor protein by sequestration of the Shine-Dalgarno sequence in a stem-loop structure upon ligand binding (55, 56). In addition, transcription termination is triggered by inhibition of translation via exposure of a Rho-utilization site, which otherwise would be shielded by translating ribosomes (54). As an independent control mechanism, the RNA chaperone Hfq is recruited to the translation initiation region via binding of the sRNA OmrA to the *btuB* CDS. Thereby, ribosome binding is blocked, which is accompanied by a degradosome-dependent decrease in the mRNA level (54). In contrast, expression control of *rpfA* encoding a muralytic enzyme of *S. coelicolor* combines two cis-acting elements, that is, a cyclic di-AMP-binding riboswitch and antisense-RNA (asRNA) Scr3097 (57). In this OFF-riboswitch, cyclic di-AMP binding to the aptamer promotes premature transcription termination. asRNA Scr3097, which completely covers the 3′-UTR of *rpfA,* was shown to positively affect *rpfA* mRNA level, possibly via mRNA stabilization (57).

In conclusion, we show that the expression of the *gcvT*-NGFG_01513-*gcvH* operon of *N. gonorrhoeae* encoding components of the GCV system involves a complex interplay of cis- and trans-acting RNA-based regulatory elements, a translational tandem riboswitch, and the two sibling sRNAs NgncR_162 and NgncR_163. While the riboswitch responds to the availability of glycine, the sibling sRNAs link the GCV system of gonococci to a different, as yet undiscovered cellular signal. Such systems, in which both a riboswitch and sRNAs in conjunction control gene expression of a single target, underline the enormous versatility of riboregulatory mechanisms in bacteria and a new way of integration of different cellular signals.

## MATERIALS AND METHODS

### Bacterial strains and growth conditions

The *N. gonorrhoeae* mutants used in this study were derived from wild-type strain MS11 (GenBank accession number NC_022240.1) and are listed in Table S1. *N. gonorrhoeae* was grown on GC agar (Oxoid) plates with 1% vitamin mix (16) for 14–16 h at 37°C in a humidified 5% CO_2_ atmosphere. Liquid cultures were grown in PPM (proteose peptone #3 [15 g], soluble starch [1 g], KH_2_PO_4_ [4 g], K_2_HPO_4_ [1 g], NaCl [5 g]/l dH_2_O) containing 1% vitamin mix and 0.04% (wt/vol) NaHCO_3_. Growth in chemically defined medium was conducted in CDM10 (58) either lacking glycine or containing 2.5 mM glycine. Media were supplemented with kanamycin, erythromycin, or spectinomycin at final concentrations of 40 µg/mL, 7 µg/mL, and 50 µg/mL, respectively, when required. *Escherichia coli* DH5α (59) was cultured in lysogeny broth (LB). When required, antibiotics were added to the following final concentrations: ampicillin, 100 µg/mL, kanamycin 30 µg/mL, and chloramphenicol, 30 µg/mL.

### Construction of *N. gonorrhoeae* mutants

All *N. gonorrhoeae* mutants were constructed by allelic exchange mutagenesis using either DNA fragments or plasmid DNA for transformation. Unless otherwise stated, chromosomal DNA of wild-type strain MS11 was used as template DNA for the synthesis of *N. gonorrhoeae*-specific fragments. PCR primers for the amplification of DNA fragments used for mutant construction are listed in Table S2. PCR products were checked by sequence analysis for proper amplification. Kanamycin-, erythromycin-, or spectinomycin-resistant transformants were screened for the desired recombination events by PCR using appropriate primer combinations.

#### MS11 gcvT-F and MS11 ΔΔgcvT-F

The DNA fragment to construct a C-terminally 3xFLAG-tagged derivative of *gcvT* was obtained via overlap extension PCR by the combination of DNA segments comprising (i) *gcvT* (amplified with primer pair gcvT-F1/gcvT-F2) and (ii) the sequence encoding 3xFLAG followed by *ermC* and the intergenic region between *gcvH* and NGFG_01515, as well as part of NGFG_01515 (amplified with primer pair gcvT-F3/gcvH-F6 using chromosomal DNA of strain MS11 gcvH-F (17) as template DNA). This DNA fragment was transformed into *N. gonorrhoeae* strains MS11 and ΔΔ162/163 to yield mutants MS11 gcvT-F and MS11 ΔΔgcvT-F.

#### MS11 gcvT-Fsm, MS11 ΔΔgcvT-Fsm, and MS11 ΔgcvT-Fsm,163m9

Strain MS11 gcvT-Fsm with mutation of the predicted sRNA interaction site of *gcvT* (GGAGAAACTTGAGAATGACTGCTC to GGAGA**TCGC**TGAGAATGAC**ACTAG**) was created via allelic exchange mutagenesis in two subsequent steps. First, *gcvT*, its immediate upstream sequence, HP_01513, and *gcvH* were deleted via transformation of MS11 with a DNA fragment comprising the *gcvT* promoter and 5′-UTR (amplified with primer pair gcvTm1/gcvTm2), as well as *aadA* and the downstream region of *gcvH* (amplified with primer pair gcvTm3/gcvH-F6 from strain MS11 RSWgcvT-F), which was assembled via overlap extension PCR. Transformation of the resulting strain, MS11 ΔgcvTH, with a DNA fragment assembled from PCR products generated with primer combinations gcvTm1/gcvTm4 and gcvTm5/gcvH-F6 (template DNA from MS11 gcvT-F), respectively, and selection for erythromycin resistance yielded mutant MS11 gcvT-Fsm carrying 3xFLAG-tagged *gcvT* with alterations in the immediate 5′-UTR and second to fourth codon. Deletion of the sibling sRNAs in strain MS11 gcvT-Fsm resulted in strain ΔΔgcvT-Fsm and was achieved by transformation with a DNA fragment amplified with primer pair Δ162-1/Δ163-4 from chromosomal DNA of mutant MS11 ΔΔ162/163 (16). Strain MS11 ΔgcvT-Fsm,163m9 harbors a deletion of NgncR_162 and expresses a NgncR_163 allele with complementarity to the gcvT-Fsm mutation in its single-stranded region 1 sequence. To create this strain, PCR fragments amplified with primer combinations Δ162-1/163m5, 163m6/163m2, and 163m3/163m7 were assembled, yielding a DNA segment consisting of the upstream region of NgncR_162 fused to the mutated NgncR_163 allele. This fragment was subsequently combined with a kanamycin resistance cassette and the downstream region of NgncR_163 (amplified with primer combination 163m8/Δ163-4 from chromosomal DNA of MS11 ΔΔ162/163). The entire DNA segment was then transformed into MS11 gcvT-Fsm to yield ΔgcvT-Fsm,163m9.

#### MS11 gcvP-F, MS11 ΔΔgcvP-F, MS11 gcvL-F, MS11 ΔΔgcvL-F, and MS11 1544-F, MS11 ΔΔ1544-F

These mutants carrying C-terminally 3xFLAG-tagged derivatives of *gcvP*, *gcvL,* and NGFG_01544 were constructed by transformation of MS11 and MS11 ΔΔ162/163 with DNA fragments comprising part of the coding sequence fused to 3xFLAG, a spectinomycin resistance cassette (*aadA*), and approximately 500 bp of the respective downstream region. DNA segments for overlap extension PCR were amplified with primers gcvP-F1 to gcvP-F6, gcvL-F1 to gcvL-F6, and 1544-F1 to 1544-F6. DNA segments comprising the 3xFLAG-sequence and *aadA1* were amplified using chromosomal DNA of strain MS11 RSWΔgcvH-F (see below) as template.

#### MS11 P*_opa_*gcvH-F and ΔΔP*_opa_*gcvH-F

In MS11 P*_opa_*gcvH-F, part of the 5′-UTR of *gcvT* (from positions −80 to −9) is inserted between the promoter of the *Neisseria opa* gene NGFG_02432 (P*_opa_*) and C-terminally 3xFLAG-tagged *gcvH*. To construct this mutant, first a spectinomycin-resistant derivative of Ng MS11 expressing *gcvH* with a C-terminal 3xFLAG-tag was created by transformation of strain MS11 with a DNA fragment amplified with primer pair gcvH-F1/gcvH-F6 from chromosomal DNA of mutant RSWgcvH-F (see below). This mutant was used as the parent strain for the transformation of a DNA fragment consisting of part of the coding and upstream region of NGFG_01511, *ermC*, 55 bp encompassing P*_opa_*, part of the 5′-UTR of *gcvT* (from positions −80 to −9), and *gcvH* (positions −13 to 384). This DNA fragment was assembled by overlap extension PCR from DNA segments amplified with primer pairs DRSW11/gcvH-F15 and gcvH-F16/gcvH-F2 from chromosomal DNA of strains MS11 RSWΔ and MS11 RSWgcvH-F, respectively (see below). Allelic exchange mutagenesis to delete the sibling sRNA genes NgncR_162 and NgncR_163 has been described previously (16) and yielded strain ΔΔP*_opa_*gcvH-F.

#### MS11 RSW, MS11 RSWΔ, MS11 ΔΔRSW, and MS11 ΔΔRSWΔ

These strains express the *gcvT*-NGFG_01513-*gcvH* operon under control of P*_opa_* and comprise either the full-length *gcvT* 5′-UTR (RSW) or a truncated 5′-UTR lacking the predicted glycine riboswitch sequence (RSWΔ). To construct these strains, three DNA fragments were sequentially combined by overlap extension PCR. First, a DNA fragment comprising *ermC* and the *Neisseria* P*_opa_* promoter was amplified from chromosomal DNA of strain MS11 P*_opa_*45 (17) with either primer combination DRSW-3/DRSW-4 or DRSW-3/DRSW-5, and this DNA segment was combined with DNA fragments comprising either the full-length or a truncated 5′-UTR, as well as part of the *gcvT* coding sequence (primer combinations DRSW-6/DRSW-8 and DRSW-7/DRSW-8, respectively). Finally, the resulting DNA fragments were fused to a DNA segment encompassing part of the intergenic region between *gcvT* and NGFG_01511 and part of the coding sequence of NGFG_01511, which was amplified with primer pair DRSW-1/DRSW-2. The combined DNA fragments were transformed into wild-type MS11 and MS11 ΔΔ162/163. Erythromycin-resistant colonies were selected, yielding mutants MS11 RSW, MS11 RSWΔ, MS11 ΔΔRSW, and MS11 ΔΔRSWΔ.

#### MS11 RSWgcvH-F, MS11 RSWΔgcvH-F, MS11 ΔΔRSWgcvH-F, and MS11 ΔΔRSWΔgcvH-F

To add a C-terminal 3xFLAG-tag to *gcvH*, mutants MS11 RSW, MS11 RSWΔ, MS11 ΔΔRSW, and MS11 ΔΔRSWΔ were transformed with a DNA fragment comprising 3xFLAG-tagged *gcvH* and 582 bp from the downstream region of *gcvH* (amplified from chromosomal DNA of MS11 gcvH-F (17) with primer pairs gcvH-F1/gcvH-F11 and gcvH-F14/gcvH-F6, respectively), which flank *aadA* (amplified with primer pair gcvH-F12/gcvH-F13).

#### MS11 RSWgcvT-F, MS11 RSWΔgcvT-F

To create mutants, RSWgcvT-F and RSWΔgcvT-F strains MS11 RSW and MS11 RSWΔ were transformed with a DNA segment consisting of the 3′-half of 3xFLAG-tagged *gcvT*, *aadA,* and 539 bp from the downstream region of *gcvH*. This fragment was obtained by a combination of PCR fragments amplified with primer pairs gcvT-F1/gcvT-F2 (template DNA from strain MS11 gcvT-F) and gcvT-F3/gcvH-F6 (template DNA from strain MS11 RSWgcvH-F) via overlap extension PCR.

#### MS11 P*_gcvT_*ΔgcvT-F

In mutant MS11 P*_gcvT_*ΔgcvT-F, 3xFLAG-tagged *gcvT* with truncated 5-UTR (lacking nucleotides corresponding to positions −19 to −242 with respect to the *gcvT* start codon) is transcribed under control of the *gcvT* promoter. To construct this strain, the *gcvT* promoter and its upstream sequence were amplified with primer pair DRSW-1/DRSW-12. The resulting PCR product was combined with a DNA fragment comprising 3xFLAG-tagged *gcvT*, *ermC,* and about 500 bp from the downstream region of *gcvH* (amplified from chromosomal DNA of strain MS11 gcvT-F with primer pair DRSW-13/gcvH-F6). This fragment was transformed into parent strain MS11 ΔgcvTH in which the *gcvT-*NGFG_01513*-gcvH* operon was substituted by *aadA*.

#### MS11 RSWgcvT-Fm1, MS11 RSWgcvT-Fm2, MS11 RSWgcvT-Fm3, MS11 RSWgcvT-Fm4, MS11 RSWm1, MS11 RSWm3, MS11 ΔΔRSWm3, MS11 RSWm4, and MS11 ΔΔRSWm4

To construct mutants RSWgcvT-Fm1 and RSWgcvT-Fm2, DNA segments comprising P*_opa_* and the *gcvT* 5′-UTR with the desired aptamer 1 and aptamer 2 mutations and part of the *gcvT* coding sequence were created via overlap extension PCR using PCR fragments amplified with primer pairs RSWmut-4/RSWmut-19 and RSWmut-20/DRSW-8 (aptamer 1) or RSWmut-4/RSWmut-17 and RSWmut-18/DRSW-8 (aptamer 2) from chromosomal DNA of strain MS11 RSW. These fragments were then combined with DNA fragments encoding part of NGFG_01511 and its upstream region (amplified with primer pair (DRSW-1/RSWmut-1) and a kanamycin resistance cassette (amplified with primer pair RSWmut-2/RSWmut-3). The resulting PCR products were then transformed into the parent strain RSWΔgcvT-F. The same strategy was applied to construct mutants RSWgcvT-Fm3 and RSWgcvT-Fm4. Here, the DNA fragments comprising the desired mutations immediately upstream of the *gcvT* start codon were created by a combination of PCR products obtained with primer pairs RSWmut-4/RSWmut-21 and RSWmut-22/DRSW-8 (m3) or RSWmut-4/RSWmut-15 and RSWmut-16/DRSW-8 (m4). The aptamer 1 mutation (m1) and 5′-UTR mutations m3 and m4 were also introduced into the RSW genetic background by transformation of the respective DNA fragments into strain MS11 RSWΔ, yielding mutants MS11 RSWm1, MS11 RSWm3, and MS11 RSWm4. Strains MS11 ΔΔRSWm3 and MS11 ΔΔRSWm4 were created by replacing the sibling sRNA genes in MS11 RSWm3 and MS11 RSWm4 with *ermC* via allelic exchange mutagenesis. The DNA segment used for transformation was assembled from PCR fragments amplified with primer pairs D162-1/D162-2(erm), 162erm5/163erm3 (template: plasmid pMR68),(60) and D163-3/D163-4, respectively.

#### MS11 RSWΔ2 and RSWΔ2m5

Mutant RSWΔ2 was created by transformation of MS11 with a DNA fragment obtained via overlap extension PCR from DNA segments amplified with primer pairs DRSW-1/DRSW-16 and DRSW-17/DRSW-8 from the chromosomal DNA of mutant MS11 RSW. To create mutant RSWΔ2m5, PCR products amplified from chromosomal DNA of strain MS11 RSWm1 with primer pair DRSW-11/RSWmut-11 and RSWmut-12/DRSWex3 were combined by overlap extension PCR. The resulting DNA fragment containing the desired mutation in the riboswitch aptamer 2 sequence was then used as a template for the amplification of DNA fragments consisting of part of the NGFG_01511 sequence and its upstream region, a kanamycin resistance cassette and the P*_opa_* promoter (amplified with primer pair DRSW-1/DRSW-18) and the truncated 5′-UTR and part of the *gcvT* gene (amplified with primer pair DRSW-19/DRSW-8), which were subsequently combined. Transformation of MS11 RSWΔ2 with the resulting DNA fragment yielded mutant MS11 RSWΔ2m5.

#### MS11 RSWgfp

Mutant RSWgfp harbors a fusion of the full-length 5′-UTR of *gcvT* with the reporter gene *gfp* (61) under the control of P*_opa_*. First, the 5′-UTR of *gcvT* and *gfp* was amplified with primer pairs RSWgfp1/RSWgfp2 and RSWgfp3/RSWgfp4 (template DNA: plasmid pKEN), the PCR products were combined via overlap extension PCR, and the resulting fragment was cloned into EcoRV/SalI-digested plasmid pAIE-162 (17). The insert was reamplified with primer pair PoRgfp-1/PoRgfp-2 and was then combined with a DNA segment comprising *aadA* and the downstream region of *gcvH* (amplified with primer pair PoRgfp-3/gcvH-F6 from chromosomal DNA of strain MS11 RSWgcvH-F). The resulting DNA fragment was transformed into strain MS11 RSW. Selection for spectinomycin-resistant colonies yielded mutant MS11 RSWgfp.

### Construction of plasmids pXG-gcvT and pXG-gcvP

Interaction of the *gcvT* and *gcvP* 5′-UTRs with the sibling sRNAs was analyzed in *E. coli* DH5α (59) using a GFP-SF-based reporter system (38). To create plasmid pXG-gcvT, a DNA fragment encompassing 73 bp of the *gcvT* 5′-UTR as well as its first 33 codons (amplified with primer pair gcvT5UTR-5/gcvT5UTR-4) was cloned into the BfrBI and NheI digested plasmid pXG10-SF (38). Plasmid pXG-gcvP was obtained by cloning of a DNA fragment amplified with primer pair gcvP5UTR-1/gcvP5UTR2 and comprising the intergenic region between NGFG_01593 and *gcvP* and encoding the last 13 amino acids of NGFG_01593 as well as the first 33 codons of *gcvP* into the intercistronic fusion vector pXG30-SF (38). Plasmids pJV-300 and pJV-162 expressing NgncR_162 have been described previously (16, 62)

### RNA preparation and real-time quantitative PCR

*N. gonorrhoeae* MS11 and ΔΔ162/163 were grown to an OD_550_ = 0.4 in modified CDM10 (0 or 2.5 mM glycine). RNA was prepared using the miRNeasy Micro Kit (Qiagen) according to the manufacturer’s instructions, followed by DNase I treatment. qRT-PCR experiments were performed as described previously (17).

### Immunoblot analysis

For the analysis of GFG expression in *E. coli,* bacteria were grown to an OD_600_ = 1.0 in LB broth. Bacteria from a culture volume of 2 mL were pelleted and resuspended in 200 µL of Laemmli buffer. *N. gonorrhoeae* was grown to an OD_550_ = 0.4 in PPM or modified CDM10 (0 or 2.5 mM glycine) medium. Cells from 1 mL of culture were harvested by centrifugation and resuspended in 50 µL of Laemmli buffer. Samples were incubated for 5 min at 95°C. Western blot analysis of the samples was performed as described previously (16). Quantification of signal intensities was performed using ImageJ (63).

## SUPPLEMENTAL MATERIAL

Supplemental figures and tables jb.00593-25-s0001.docx Tables S1 and S2, and Figures S1 to S8.

ASM does not own the copyrights to Supplemental Material that may be linked to, or accessed through, an article. The authors have granted ASM a non-exclusive, world-wide license to publish the Supplemental Material files. Please contact the corresponding author directly for reuse.
