# Supplementary material for: Complex interplay of riboregulatory mechanisms at the gcvT-NGFG_01513-gcvH operon of Neisseria gonorrhoeae
Source: J Bacteriol. 2026 Mar 5;208(4):e00593-25. doi: 10.1128/jb.00593-25 (PMC13104608; doi:10.1128/jb.00593-25)
Supplement: Supplemental figures and tables — Tables S1 and S2, and Figures S1 to S8. [file jb.00593-25-s0001.docx]

**SUPPLEMENTARY MATERIAL**

**Complex interplay of riboregulatory mechanisms at the *gcvT*-NGFG_01513-*gcvH* operon of *Neisseria gonorrhoeae***

Susanne Bauer, Thomas Rudel and Dagmar Beier

Chair of Microbiology, Theodor-Boveri-Institute, University of Würzburg, Würzburg, Germany

Table of Contents:

Tab. S1: Bacterial strains and plasmids used in this study

Tab. S2: Oligonucleotides used in this study

Fig. S1: *In silico* prediction of sRNA-mRNA interactions

Fig. S2: Transcript analysis of NGFG_01593-*gcvP* and *gcvT-*NGFG_01513*-gcvH* by RT-PCR

Fig. S3: Quantification of *gcvT*-specific transcripts in strains MS11 gcvT-F and MS11 gcvH-F

Fig. S4: Expression of translational *gcvT*- and *gcvP*-*gfp* fusions in *E. coli* in presence and absence of sRNA

NgncR_162

Fig. S5: Growth of *N. gonorrhoeae* MS11 in chemically defined CDM10 medium in the absence and

presence of glycine

Fig. S6: Conservation of the *gcvT* tandem glycine riboswitch in the genus *Neisseria*

Fig. S7: Immunoblot analysis of *N. gonorrhoeae* MS11 gcvT-F and MS11 P*_gcvT_*ΔgcvT-F with truncated *gcvT*

5’-UTR

Fig. S8: Glycine-responsive expression of 3xFLAG-tagged *gcvT* in *N. gonorrhoeae* strains MS11

RSWgcvT-F and MS11 RSWΔgcvT-F in the absence and presence of bicyclomycin

**Table S1: Bacterial strains and plasmids used in this study**

| Strain / plasmid | description | reference |
| --- | --- | --- |
|  |  |  |
| *E. coli* |  |  |
| DH5α | F- gyrA96 (Nalr) recA1 relA1 endA1 thi-1 hsdR17(rk-mk+) glnV44 deoR(lacZYA-argF)U169 [Φ80d(lacZ)M15] | Thermo Fisher Scientific |
|  |  |  |
| *N. gonorrhoeae* |  |  |
| MS11 | wild-type *N. gonorrhoeae* | laboratory strain  collection |
| MS11 162/163 | MS11 with sRNA genes NgncR_162 and NgncR_163 substituted by a kanamycin resistance cassette | (16) |
| MS11 gcvT-F | MS11 expressing C-terminally 3xFLAG-tagged GcvT (with deletion of NGFG_01513 and *gcvH*) | this study |
| MS11 gcvT-F | MS11 162/163 expressing C-terminally 3xFLAG-tagged GcvT (with deletion of NGFG_01513 and *gcvH*) | this study |
| MS11 ΔgcvTH | MS11 with *gcvT* including its immediate upstream sequence, HP_01513 and *gcvH* substituted by a spectinomycin resistance cassette | this study |
| MS11 gcvT-Fsm | MS11 encoding C-terminally 3xFLAG-tagged *gcvT* with mutations in the immediate upstream sequence and codons 2-4 | this study |
| MS11 gcvT-Fsm | MS11 162/163 encoding C-terminally 3xFLAG-tagged *gcvT* with mutations in the immediate upstream sequence and within codons 2-4 | this study |
| MS11 ΔgcvT-Fsm,163m9 | MS11 gcvT-Fsm with deletion of NgncR_162 and encoding a NgncR_163 allele with mutated SSR1 sequence (complementary to the gcvT-Fsm 5’-UTR mutation) | this study |
| MS11 gcvP-F | MS11 expressing C-terminally 3xFLAG-tagged GcvP (NGFG_01592) | this study |
| MS11 gcvP-F | MS11 162/163 expressing C-terminally 3xFLAG-tagged GcvP | this study |
| MS11 gcvL-F | MS11 expressing C-terminally 3xFLAG-tagged GcvL (NGFG_0807) | this study |
| MS11 gcvL-F | MS11 162/163 expressing C-terminally 3xFLAG-tagged GcvL | this study |
| MS11 1544-F | MS11 expressing C-terminally 3xFLAG-tagged NGFG_01544 | this study |
| MS11 1544-F | MS11 162/163 expressing C-terminally 3xFLAG-tagged NGFG_01544 | this study |
| MS11 P*_opa_*gcvH-F | MS11 expressing C-terminally 3xFLAG-tagged GcvH under control of the promoter of a neisserial *opa* gene and fused to a truncated *gcvT* 5’-UTR | this study |
| MS11 P*_opa_*gcvH-F | MS11 162/163 expressing C-terminally 3xFLAG-tagged GcvH under control of the promoter of a neisserial *opa* gene (P*_opa_*) and fused to a truncated *gcvT* 5’-UTR | this study |
| MS11 RSW | MS11 with substitution of the *gcvT* promoter by P*_opa_* | this study |
| MS11 ΔΔRSW | MS11 162/163 with substitution of the *gcvT* promoter by P*_opa_* |  |
| MS11 RSWΔ | MS11 with substitution of the *gcvT* promoter by P*_opa_* and truncated *gcvT* 5’-UTR (fusion of P*_opa_* to position -80 of *gcvT* 5’-UTR) | this study |
| MS11 ΔΔRSWΔ | MS11 162/163 with substitution of the *gcvT* promoter by P*_opa_* and truncated *gcvT* 5’-UTR | this study |
| MS11 RSWΔ2 | MS11 with substitution of the *gcvT* promoter by P*_opa_* and truncated *gcvT* 5’-UTR (fusion of P*_opa_* to position -98 of *gcvT* 5’-UTR) | this study |
| MS11 RSWΔ2m5 | MS11 RSWΔ2 with AATATT mutation in the *gcvT* 5’-UTR (position -81 to -76) | this study |
| MS11 RSWgcvH-F | MS11 RSW expressing C-terminally 3xFLAG-tagged GcvH | this study |
| MS11 ΔΔRSWgcvH-F | MS11 ΔΔRSW expressing C-terminally 3xFLAG-tagged GcvH | this study |
| MS11 RSWΔgcvH-F | MS11 RSWΔ expressing C-terminally 3xFLAG-tagged GcvH | this study |
| MS11 ΔΔRSWΔgcvH-F | MS11 ΔΔRSWΔ expressing C-terminally 3xFLAG-tagged GcvH | this study |
| MS11 RSWgcvT-F | MS11 RSW expressing C-terminally 3xFLAG-tagged GcvT (with deletion of NGFG_01513 and *gcvH*) | this study |
| MS11 RSWΔgcvT-F | MS11 RSWΔ expressing C-terminally 3xFLAG-tagged GcvT (with deletion of NGFG_01513 and *gcvH*) | this study |
| MS11 RSWgfp | MS11 with *gfp* transcribed under control of P*_opa_* and fused to the *gcvT* 5’-UTR, deletion of NGFG_01513 and *gcvH* | this study |
| MS11 RSWm1 | MS11 RSW with mutation in the glycine binding pocket of tandem glycine riboswitch aptamer 1 (U67 to G) | this study |
| MS11 RSWm3 | MS11 RSW with mutation of nucleotides corresponding to position -2 to -5 of the *gcvT* 5’-UTR (TGAG to GACA) | this study |
| MS11 ΔΔRSWm3 | MS11 RSWm3 with deletion of NgncR162/163 | this study |
| MS11 RSWm4 | MS11 RSW with mutation of nucleotides corresponding to position -1 to -10 of the *gcvT* 5’-UTR (AAACTTGAGA to CTCACCACAT) | this study |
| MS11 ΔΔRSWm4 | MS11 RSWm4 with deletion of NgncR162/163 | this study |
| MS11 RSWgcvT-Fm1 | MS11 RSWgcvT-F with mutation in the glycine binding pocket of tandem glycine riboswitch aptamer 1 (U67 to G) | this study |
| MS11 RSWgcvT-Fm2 | MS11 RSWgcvT-F with mutation in the glycine binding pocket of tandem glycine riboswitch aptamer 2 (U224 to G) | this study |
| MS11 RSWgcvT-Fm3 | MS11 RSWgcvT-F with mutation of nucleotides corresponding to position -2 to -5 of the *gcvT* 5’-UTR (TGAG to GACA) | this study |
| MS11 RSWgcvT-Fm4 | MS11 RSWgcvT-F with mutation of nucleotides corresponding to position -1 to -10 of the *gcvT* 5’-UTR (AAACTTGAGA to CTCACCACAT) | this study |
| MS11 P*_gcvT_*ΔgcvT-F | MS11 expressing FLAG-tagged *gcvT* with truncated 5’-UTR under control of the *gcvT* promoter | this study |
|  |  |  |
| plasmids |  |  |
|  |  |  |
| pMR68 | complementation plasmid for *N. gonorrhoeae* and *N. meningitidis* | (60) |
| pJV300 | plasmid expressing a nonsense sRNA under control of the P_LlacO_ promoter | (62) |
| pXG10-SF | superfolder *gfp*-based translational fusion plasmid | (38) |
| pXG30-SF | superfolder *gfp*-based translational fusion plasmid for intercistronic fusions | (38) |
| pJV-162 | derivative of pJV300 expressing NgncR_162 | (17) |
| pXG-gcvT | pXG10-SF expressing a fusion of *gfp* to a DNA segment comprising 73 bp of the *gcvT* 5’ UTR and the first 33 codons of *gcvT* | this study |
| pXG-gcvP | pXG30-SF harbouring a DNA-fragment comprising the last 13 codons of NGFG_01593, the intergenic region and the first 13 codons of *gcvP* | this study |

**Tab. S2:** **Oligonuncleotides used in this study**

| Name | Sequence (5‘ to 3‘)^a^ | amplification of |
| --- | --- | --- |
|  |  |  |
| gcvT5UTR-5 | tataatatgcatCAAGGACAGATAGGGTCATC | region comprising 73 nucleotides of the 5’-UTR and the N-terminal 33 codons of *gcvT* |
| gcvT5UTR-4 | tataatgctagcACCATAATGGATGGGCAGTTC | region comprising 73 nucleotides of the 5’-UTR and the N-terminal 33 codons of *gcv*T |
| gcvP5UTR-1 | tataatatgcatCACCCTTGCCCGAATCATCC | region comprising the C-terminal 13 codons of NGFG_01593, intergenic region and N-terminal 33 codons of *gcvP* |
| gcvP5UTR-2 | tataatgctagcCATGCTCTTCTCGCCGACAGC | region comprising the C-terminal 13 codons of NGFG_01593, intergenic region and N-terminal 33 codons of *gcvP* |
| gcvT-F1 | gccgtctgaaTGGGCAACGACTGGTTTGTCG | *gcvT* |
| gcvT-F2 | CCGTCATGGTCTTTGTAGTCATCAAACTGTTTTTGACCGCTGC | *gcvT* with 3’-overhang to 3xFLAG sequence |
| gcvT-F3 | GCAGCGGTCAAAAACAGTTTGATGACTACAAAGACCATGACGG | 3xFLAG, *ermC* and downstream region of *gcvH* with 5’-overhang to *gcvT* |
| gcvH-F6 | CATACTTTCCTGTTCTTGAGCC | downstream region of *gcvH* |
| gcvP-F1 | gccgtctgaaCGCTGGGCATTGCTCAACG | *gcvP* |
| gcvP-F2 | ACCGTCATGGTCTTTGTAGTCGTCTTCATAATTTTCCATCGGC | *gcvP* with 3’-overhang to 3xFLAG sequence |
| gcvP-F3 | GCCGATGGAAAATTATGAAGACGACTACAAAGACCATGACGGT | 3xFLAG and *aadA* with 5’-overhang to *gcvP* |
| gcvP-F4 | TGTCTTCGGGGTTTCAACAGT TTATTTGCCGACTACCTTGGTG | 3xFLAG and *aadA* with 3’-overhang to downstream region of *gcvP* |
| gcvP-F5 | CACCAAGGTAGTCGGCAAATAAACTGTTGAAACCCCGAAGACA | downstream region of *gcvP* with 5’-overhang to *aadA* |
| gcvP-F6 | GAAAGGGCAAATGGCGAAAGC | downstream region of *gcvP* |
| gcvL-F1 | gccgtctgaaCATCAATATCGACAAAGACGC | *gcvL* |
| gcvL-F2 | ACCGTCATGGTCTTTGTAGTCGGCTTTCAATTTCGCATCGGC | *gcvL* with 3’-overhang to 3xFLAG sequence |
| gcvL-F3 | GCCGATGCGAAATTGAAAGCCGACTACAAAGACCATGACGGT | 3xFLAG and spec^r^ with 5’-overhang to *gcvL* |
| gcvL-F4 | GGCATTGTTTTGCCATATCGGTTATTTGCCGACTACCTTGGTG | 3xFLAG and spec^r^ with 3’-overhang to downstream region of *gcvL* |
| gcvL-F5 | CACCAAGGTAGTCGGCAAATAACCGATATGGCAAAACAATGCC | downstream region of *gcvL* with 5’-overhang to *aadA* |
| gcvL-F6 | TCAGCTCAAGGCCTTTGTGC | downstream region of *gcvP* |
| 1544-F1 | gccgtctgaaGAAGCAACGGCAGACATTCC | NGFG_01544 |
| 1544-F2 | ACCGTCATGGTCTTTGTAGTCACCGAACAACCAGTTTTCCAAC | NGFG_01544 with 3’-overhang to 3xFLAG sequence |
| 1544-F3 | GTTGGAAAACTGGTTGTTCGGTGACTACAAAGACCATGACGGT | 3xFLAG and spec^r^ with 5’-overhang to NGFG_01544 |
| 1544-F4 | TGTGCAATGCCGTCTGAAAGTTTATTTGCCGACTACCTTGGTG | 3xFLAG and spec^r^ with 3’-overhang to downstream region of NGFG_01544 |
| 1544-F5 | CACCAAGGTAGTCGGCAAATAAACTTTCAGACGGCATTGCACA | downstream region of NGFG_01544 with 5’-overhang to *aadA* |
| 1544-F6 | GCTTGAAGGTGTGGTTTAAACG | downstream region of NGFG_01544 |
| DRSW-1 | gccgtctgaaATGCCTGAAGCAGGTAGTCG | part of NGFG_01511 |
| DRSW-2 | CGCAATTAACCCTCACTAAAGATGTCTTGAATCGGCGGTTTGG | upstream region of NGFG_01511 with 3’-overhang to *ermC* |
| DRSW-3 | CCAAACCGCCGATTCAAGACATCTTTAGTGAGGGTTAATTGCG | *ermC* with 5’-overhang to upstream region of NGFG_01511 |
| DRSW-4 | ACACTCTCCTGCACATTTCCCATTATATCGGGTTCCGGGCG | *opa* promoter with 3’-overhang to full length 5’-UTR of *gcvT* |
| DRSW-5 | CCTATCTGTCCTTGAACCTGAATTATATCGGGTTCCGGGCG | *opa* promoter with 3’-overhang to truncated 5’-UTR of *gcvT* |
| DRSW-6 | CGCCCGGAACCCGATATAATGGGAAATGTGCAGGAGAGTGT | full length 5’-UTR of *gcvT* with 5’-overhang to *opa* promoter |
| DRSW-7 | CGCCCGGAACCCGATATAATTCAGGTTCAAGGACAGATAGG | truncated 5’-UTR of *gcvT* with 5’-overhang to *opa* promoter |
| DRSW-8 | AATGGCTTTAGGACCTTGTACG | part of *gcvT* |
| DRSW-11 | gccgtctgaaTGGTGGGATAAAAGCGTATCC | part of NGFG_01511 |
| DRSW-12 | CATTCTCAAGTTTCTCCGGATTTTAAGGGTGTCTGCGCCTTCG | *gcvT* promoter with 3’-overhang to *gcvT* RBS and CDS |
| DRSW-13 | CGAAGGCGCAGACACCCTTAAAATCCGGAGAAACTTGAGAATG | *gcvT* RBS and CDS with 5’-overhang to *gcvT* promoter |
| DRSW-16 | CTGAGATTTTCGACCGTATCCGATTATATCGGGTTCCGGGCGG | *opa* promoter with 3’-overhang to truncated 5’-UTR of *gcvT* |
| DRSW-17 | CCGCCCGGAACCCGATATAATCGGATACGGTCGAAAATCTCAG | truncated 5’-UTR of *gcvT* with 5’-overhang to *opa* promoter |
| DRSW-18 | GAAAATATTATTTTCGACCGTATCCGATTATATCGGGTTCCGGGCGG | *opa* promoter with 3’-overhang to truncated/mutated 5’-UTR of *gcvT* |
| DRSW-19 | CCGCCCGGAACCCGATATAATCGGATACGGTCGAAAATAATATTTTC | truncated /mutated 5’-UTR of *gcvT* with 5’-overhang to *opa* promoter |
| DRSWex3 | AAATCCGCGCCTTGGAACG | part of *gcvT* |
| gcvTm1 | gccgtctgaaCAGTTCGACATTGGTCAGCC | *gcvT* promoter and upstream region |
| gcvTm2 | TGGCGGATTAACAAAAACCGGCCTATCTGTCCTTGAACCTGA | *gcvT* 5’-UTR with 3’-overhang to *aadA* |
| gcvTm3 | TCAGGTTCAAGGACAGATAGGCCGGTTTTTGTTAATCCGCCA | *aadA* with 5’-overhang to *gcvT* 5’-UTR |
| gcvTm4 | TTTCACTAGTGTCATTCTCAGCGATCTCCGGATTTTTTGTTCAGATGC | *gcvT* 5’-UTR and 5’-end of coding region with sm mutation |
| gcvTm5 | GGAGATCGCTGAGAATGACACTAGTGAAAACCACCCCGTTTCATCA | part of *gcvT* with sm mutation |
| gcvH-F1 | gccgtctgaaACTGCCTTCCGCCCAAAAGC | part of *gcvH* |
| gcvH-F2 | CACCGTCATGGTCTTTGTAGTC ATCCACTTCGCCCGCGTATTG | *gcvH* with 3’-overhang to 3xFLAG sequence |
| gcvH-F11 | GGCGGATTAACAAAAACCGG TTATTTATCGTCGTCATCTTTGTAGTCG | *gcvH*-3F with 3’-overhang to *aadA* |
| gcvH-F12 | ACTACAAAGATGACGACGATAAATAA CCGGTTTTTGTTAATCCGCCA | *aadA* with 5’-overhang to *gcvH*-3F |
| gcvH-F13 | GCAGTTTATGCATCCCTTAACTTATTTGCCGACTACCTTGGTG | *aadA* with 3’-overhang to downstream region of *gcvH* |
| gcvH-F14 | CACCAAGGTAGTCGGCAAATAAGTTAAGGGATGCATAAACTGC | downstream region of *gcvH* with 5’-overhang to *aadA* |
| gcvH-F15 | GTTGCTCATGGTTTGATTCTCCGGATTTTTTGTTCAGATGCCG | 5’-UTR of *gcvT* with 3’-overhang to *gcvH* |
| gcvH-F16 | CGGCATCTGAACAAAAAATCCGGAGAATCAAACCATGAGCAAC | *gcvH* with 5’-overhang to 5’-UTR of *gcvT* |
| RSWgfp-1 | tataatgatatc**T**GTGCAGGAGAGTGTTACACC | 5’-UTR of *gcvT* |
| RSWgfp-2 | GAAAAGTTCTTCTCCTTTACTCATTCTCAAGTTTCTCCGGATTT | 5’-UTR of *gcvT* with 3’-overhang to *gfp* |
| RSWgfp-3 | AAATCCGGAGAAACTTGAGAATG AGTAAAGGAGAAGAACTTTTC | *gfp* with 5’-overhang to 5’-UTR of *gcvT* |
| RSWgfp-4 | tataatgtcgacAAAACAGCCAAGCTTGCATGC | *gfp* |
| RSWmut-1 | TGAGACACAATTCATCGATGATATGTCTTGAATCGGCGGTTTGG | upstream region of NGFG_01511 with 3’-overhang to P*_opa_* |
| RSWmut-2 | CCAAACCGCCGATTCAAGACATATCATCGATGAATTGTGTCTCAA | kan^r^ with 5’-overhang to upstream region of NGFG_01511 |
| RSWmut-3 | TGGCGGATTAACAAAAACCGGCTGAAGCTTGCATGCCTGCA | kan^r^ with 3’-overhang to P*_opa_* |
| RSWmut-4 | TGCAGGCATGCAAGCTTCAGCCGGTTTTTGTTAATCCGCCA | P*_opa_*-promoter with 5’-overhang to kan^r^ |
| RSWmut-11 | CCTATCTGTCCTTGAAAATATTATTTTCGACCGTATCCGCATGATG | part of *gcvT* 5’-UTR with Δ2m3 mutation |
| RSWmut-12 | GGATACGGTCGAAAATAATATTTTCAAGGACAGATAGGGTCATC | part of *gcvT* 5’-UTR with Δ2m3 mutation |
| RSWmut-15 | GGGTGGTTTTCAGAGCAGTCATATGTGGTGAGCTCCGGATTTTTT | part of *gcvT* 5’-UTR with m4 mutation |
| RSWmut-16 | AAAAAATCCGGAGCTCACCACATATGACTGCTCTGAAAACCACCC | part of *gcvT* 5’-UTR with m4 mutation |
| RSWmut-17 | CCCTATCTGTCCTTGAACCTGCGATTTTCGACCGTATCCGCATG | part of *gcvT* 5’-UTR with m2 mutation |
| RSWmut-18 | CATGCGGATACGGTCGAAAATCGCAGGTTCAAGGACAGATAGGG | part of *gcvT* 5’-UTR with m2 mutation |
| RSWmut-19 | TGTGCAGTCCCTGATACCTGCGCGATTTAAGGGTGTCTGCG | part of *gcvT* 5’-UTR with m1 mutation |
| RSWmut-20 | CGCAGACACCCTTAAATCGCGCAGGTATCAGGGACTGCACA | part of *gcvT* 5’-UTR with m1 mutation |
| RSWmut-21 | GGGTGGTTTTCAGAGCAGTCATTTGTCAGTTTCTCCGGATTTTTTGTTC | part of *gcvT* 5’-UTR with m3 mutation |
| RSWmut-22 | ATCCGGAGAAACTGACAAATGACTGCTCTGAAAACCACCC | part of *gcvT* 5’-UTR with m3 mutation |
| PoRgfp-1 | gccgtctgaaTGTGCAGGAGAGTGTTACACC | 5’-UTR of *gcvT* |
| PoRgfp-2 | TGGCGGATTAACAAAAACCGGAAAACAGCCAAGCTTGCATGC | *gfp* with 3’-overhang to *aadA* |
| PoRgfp-3 | GCATGCAAGCTTGGCTGTTTTCCGGTTTTTGTTAATCCGCCA | *aadA* with 5’-overhang to *gfp* |
| Δ162-1 | gccgtctgaaGGCGATTTGTCCGCACAATG | upstream region of NgncR_162 |
| Δ163-4 | CCGTATCCCGATGACGGAGCT | downstream region of NgncR_163 |
| 163m2 | GGAGAAATAGAGGAGATCGCATTAACTGACTACTCGAACCAG | part of NgncR_163 with m9 mutation |
| 163m3 | GTCAGTTAATGCGATCTCCTCTATTTCTCCTTTGTAGACTTG | part of NgncR_163 with m9 mutation |
| 163m5 | GCGGAGATAAAAAATGCACACATATCCGCCGTTTCTTATCTGCTG | upstream region of NgncR_162 with overhang to the NgncR_163 promoter |
| 163m6 | CAGCAGATAAGAAACGGCGGATATGTGTGCATTTTTTATCTCCGC | NgncR_163 promoter with overhang to the upstream region of NgncR_162 |
| 163m7 | TTGAGACACAATTCATCGATGATATAACAACATCACGCACAGAGG | downstream region of NgncR_163 with overhang to kan^r^ |
| 163m8 | CCTCTGTGCGTGATGTTGTTATATCATCGATGAATTGTGTCTCAA | kan^r^ with overhang to downstream region of NgncR_163 |
| Δ162-2(erm) | CGCAATTAACCCTCACTAAAGGGAAATCCGCCGTTTCTTATCTGCTG | upstream region of NgncR_162 with 3’-overhang to *ermC* |
| Δ163-3(erm) | CCTTAACTTGTTTTTCGTGTACCTCTGTGCGTGATGTTGTTATGTTTCAT | downstream region of NgncR_163 with 5’- overhang to *ermC* |
| 162erm5 | CAGCAGATAAGAAACGGCGGATTTCCCTTTAGTGAGGGTTAATTGCG | *ermC* with 5’-overhang to upstream region of NgncR_162 |
| 163erm3 | ATGAAACATAACAACATCACGCACAGAGGTACACGAAAAACAAGTTAAGG | *ermC* with 3’-overhang to downstream region of NgncR_163 |
| 5S FW | CGGCCATAGCGAGTTGGT | amplicon for 5S RNA transcript quantification |
| 5S RV | TTGGCAGTGACCTACTTTCG | amplicon for 5S RNA transcript quantification and Northern Blot probe for 5S RNA |
| qRT1512-1 | AAACAAGTCGGTTTGCTGCT | amplicon for NGFG_01512 (*gcvT*) transcript quantification |
| qRT1512-2 | GCGATGGCGATAGATTGTTT | amplicon for NGFG_01512 (*gcvT*) transcript quantification |
| qRT1514-1 | CCGTCGGTATTACCCATCAC | amplicon for NGFG_01514 (*gcvH*) transcript quantification |
| qRT1514-2 | TGCGGCTTTTACATACTCAA | amplicon for NGFG_01514 (*gcvH*) transcript quantification |
| qRT1592-1 | GGTTTCAGACGGCCTCAAAG | amplicon for NGFG_01592 (*gcvP*) transcript quantification |
| qRT1592-1 | TGAGTATTGTTGACGCTGCG | amplicon for NGFG_01592 (*gcvP*) transcript quantification |
| qRT807-1 | CATTGCATACCGACCCCTTC | amplicon for NGFG_00807 (*gcvL*) transcript quantification |
| qRT807-2 | CAGGCCATTCTTCCACATCG | amplicon for NGFG_00807 (*gcvL*) transcript quantification |
| qRT1544-1 | GTCGGCGACCATATTTACGG | amplicon for NGFG_01544 transcript quantification |
| qRT1544-2 | AGTCCGAGCAATGTGGTGTA | amplicon for NGFG_01544 transcript quantification |
| qRT863-1 | GTGTGATTTTGTGCCGTGAC | amplicon for NGFG_00863 (*glyA*) transcript quantification |
| qRT863-2 | CGCTTCTTTAAACGCTACGG | amplicon for NGFG_00863 (*glyA*) transcript quantification |
| qRT1721-1 | AAAAGGCTTGGGCAAAAACT | amplicon for NGFG_01721 transcript quantification |
| qRT1721-2 | ATACCGAAGCTGGTTTGCAC | amplicon for NGFG_01721 transcript quantification |
| qRTgfp-1 | GGTGATGCAACATACGGAAA | amplicon for *gfp* transcript quantification |
| qRTgfp-2 | CTGGGTATCTCGCAAAGCAT | amplicon for *gfp* transcript quantification |
| RTgcvP-1 | CTGCAGGTTTCATGGTTTTGG | part of NGFG_01593 and *gcvP* |
| RTgcvP-2 | AAGTCGTCCATGCTCTTCTCG | part of NGFG_01593 and *gcvP* |
| RT1513-1 | CAGCCTGAAACAATCTATCGC | part of *gcvT* and NGFG_01513 |
| RT1513-2 | TATTCCCCGTCTATTACCACC | part of *gcvT* and NGFG_01513 |
| RTgcvH-1 | CCCAAAAGCAAGAATTGGAA | part of NGFG_01513 and *gcvH* |
| RTgcvH-2 | ACCACACCGGACTGTTCTTC | part of NGFG_01513 and *gcvH* |

1. Sequences introduced for cloning purposes are given in lower case letters. Restriction sites are underlined.

A.

B.

C.

D.

**Fig. S1:**

*In silico* prediction of sRNA-mRNA interactions. Regions of complementarity between the sibling sRNAs and their putative target genes *gcvT* (A), *gcvP* (B), *gcvL* (C) and NGFG_01544 (D) were analysed with IntaRNA (37). Numbers refer to the nucleotide positions with respect to the translational start site (+1) in the case of mRNAs and the transcription initiation site in the case of NgncR_162 or NgncR_163. The start codon is marked in bold, the RBS is underlined. Energy scores (E [kcal/mol]) from IntaRNA analysis are indicated. Predicted hybridization of sRNA NgncR_162 to the *gcvT* mRNA covers only nucleotides at position -14 to -8 in the 5’-UTR (E= -6.68 kcal/mol). Nucleotides shown in blue above the *gcvT* sequence and marked by arrows indicate mutations introduced in strain MS11 gcvT-Fsm to compromise sRNA-mRNA interaction. Nucleotides shown in blue below the sRNA sequence and marked by arrows denote complementary mutations introduced in the NgncR_163 allele of strain MS11 ΔgcvT-Fms,163m9 which should restore sRNA-mRNA hybridization in the 5’-UTR. In case of *gcvP* the region of complementarity to sRNA NgncR_163 is more extended and covers nucleotides -22 to +31 in the mRNA (E= -16.78 kcal/mol). Multiple and differing sRNA-mRNA interactions were predicted within the coding region of NGFG_01544 for both NgncR_162 and NgncR_163. The interactions with the highest energy scores are depicted in the figure.


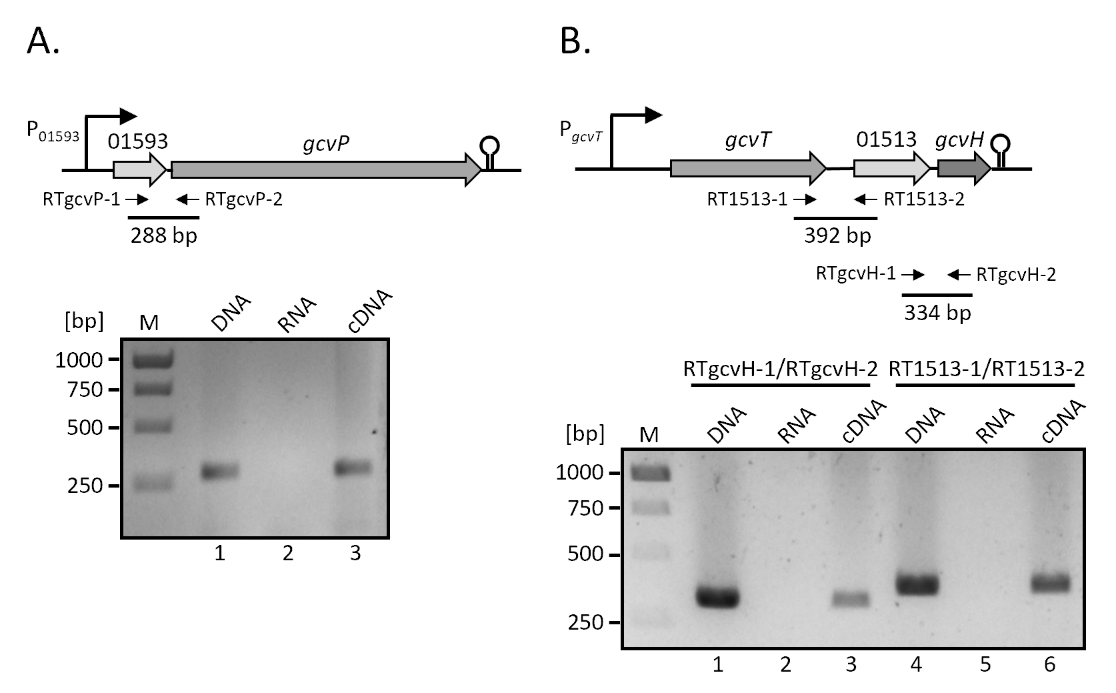


**Fig. S2:**

Transcript analysis of NGFG_01593-*gcvP* and *gcvT-*NGFG_01513*-gcvH* by RT-PCR. A schematic representation of the NGFG_01593-*gcvP* (A) and *gcvT*-NGFG_01513-*gcvH* (B) gene loci is shown. Small arrows indicate the positions of the PCR primers and the size of the respective amplicon is indicated below. PCR-reactions were performed with chromosomal DNA, RNA and cDNA obtained by reverse transcription using random priming. The results of agarose gel electrophoresis of the reaction mixtures are shown. M denotes the DNA size marker.


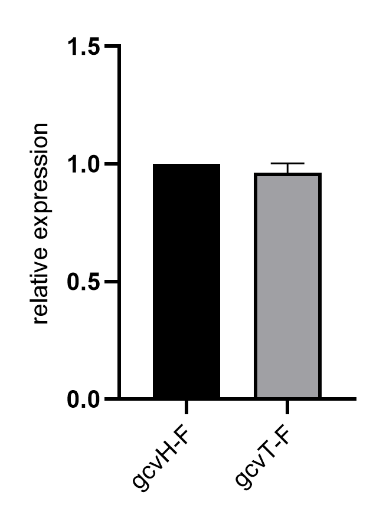


**Fig. S3:**

Quantification of *gcvT*-specific transcripts in strains MS11 gcvT-F and MS11 gcvH-F. qRT-PCR was performed on RNA extracted from strains MS11 gcvT-F and MS11 gcvH-F grown to OD_550_ = 0.4 in PPM medium. The ratio (fold-change) of the transcript amount in strain MS11 gcvT-F (carrying a deletion of NGFG_01513 and *gcvH*) relative to mutant MS11 gcvH-F (harbouring the full-length *gcvT*-HP01513-*gcvH* operon; normalized to 1) is depicted. The indicated ratio represents the mean of the results of qRT-PCR experiments performed in triplicate on cDNAs obtained from three independent RNA preparations. Error bars indicate the standard deviation.


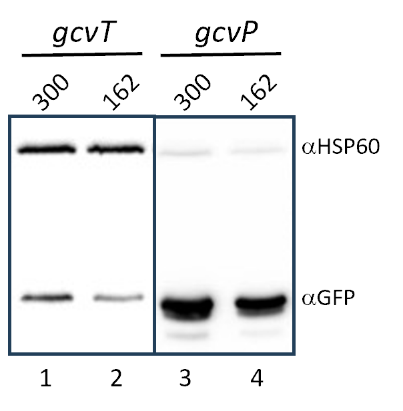


**Fig. S4:**

Expression of translational *gcvT*- and *gcvP*-*gfp* fusions in *E. coli* in presence and absence of sRNA NgncR_162. *E. coli* DH5α cells harbouring plasmid pXG-gcvT (expressing a translational *gcvT*-*gfp* fusion) or pXG-gcvP (expressing a translational *gcvP*-*gfp* fusion) were co-transformed with plasmid pJV300 expressing a nonsense sRNA (lanes 1 and 3) or plasmid pJV-162 expressing NgncR_162 (lanes 2 and 4). Protein lysates of *E. coli* cultures with OD_600_ = 1.0 were prepared, equal amounts of protein were separated on a 12% polyacrylamide gel and Western blot analysis was performed with monoclonal antibodies directed against GFP and HSP60 used as loading control. The figure shows the results from representative experiments (*gcvT*, n = 3; *gcvP*, n =2). Samples in lanes 1, 2 and 3, 4 were run on separate gels.


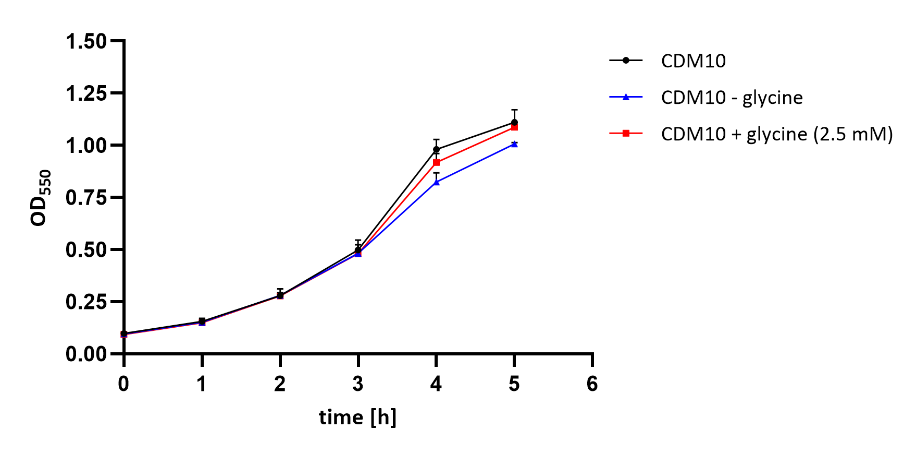


**Fig. S5:**

Growth of *N. gonorrhoeae* MS11 in chemically defined CDM10 medium in the absence and presence of glycine. Wild-type strain MS11 was grown at 37°C in PPM medium to an optical density OD_550_ = 0.4. Gonococci were sedimented by centrifugation, bacteria were resuspended in 1 ml of standard CDM10 medium and the optical density of the inoculum was determined. Standard CDM10 medium (0.3 mM glycine), CDM10 lacking glycine and CDM10 containing 2.5 mM glycine was inoculated to OD_550_ = 0.1 and the incubation was continued for 5 h. The optical density was monitored every hour. Data points represent the mean of two independent experiments.

Nani ATGTGCAGGAGAGTGTTACACCTTGCTACACGG--TGTGG------CCACCGAAGGCGCA 52

Nmu ATGTGCAGGAGAGAGCTACACCCAACTACA-----TGTGG------CCACCGAAGGCGCA 49

Nla ATGTGCAGGAGAGTGTTACACCCGATTACAA----TGTAA------CCACCGAAGGCGCA 50

Nsi ATGTGCAGGAGAGTGTTACACCCAACTACAA----TGTAA------CCACCGAAGGCGCA 50

Nme ATGTGCAGGAGAGTGTTACACCCAACTACAA----TGTAA------CCACCGAAGGCGCA 50

Ngo ATGTGCAGGAGAGTGTTACACCCAACTACAA----TGTAA------CCACCGAAGGCGCA 50

Nsu ATGTGCAGGAGAGTGTTACACCCAACTACAA----TGTAA------CCACCGAAGGCGCA 50

Npo ATGTGCAGGAGAGAGCTACACCCGATTACGC-----GTA------GCCGCCGAAGGCGCA 49

Nfl ATGTGCAGGAGAGAGCTACATCAGGTTACGC-----GTA------GCCGCCGAAGGCGCA 49

Nci ATGTGCAGGAGAGAGCTACATCAGGTTACGC-----GTA------GCCGCCGAAGGCGCA 49

Nma ATGTGCAGGAGAGAGCTACATCAGGTTACA-----TGTA------GCCGCCGAAGGCGCA 49

Nar ATATGCAGGAGAGAGT-GCGCCCG-T-------------CGGCGTGCCACCGAAGGCGCA 45

Nca CCGTGCAGGAGAGCGTTACACCCGATTACACC----GTAA------CCGCCGAAGGCGCA 50

Nwa CCGTGCAGGAGAGCGTTACACCCGATTACACC----GTAA------CCGCCGAAGGCGCA 50

Nde GTGTGCAGGAGAGGGCTACATCGGATTATA------GTG------GCCGCCGAAGGCGCA 48

Nwe GAATGCAGGAGAGTGCTACACCGTATTAC---------ACGGCAAGCCACCGAAGGCGCA 51

Nano CGATGCAGGAGAGCGCTACACCGTACTACATCAACTGTACGGCAAGCCGCCGAAGGCGCA 60

Nzo CGATGCAGGAGAGCGCTACACCGTACTACATCAACTGTACGGCAAGCCGCCGAAGGCGCA 60

********** * ** ***********

Nani GATGCCCTTAAATCGCTCAGGTAAAAGG-ACTGCACATTGAAAGAAAA-CATCTGGAGAG 110

Nmu GACACCCTTAAATCGCTCAGGTATCAGGGACTGCACATTGAAACAAAC-AATCTGGAGAG 108

Nla GACACCCTTAAATCGCTCAGGTATCAGGGACTGCACATTGAAACAAAC-AATCTGGAGAG 109

Nsi GACACCCTTAAATCGCTCAGGTATCAGGGACTGCACATTGAAACAAAC-AATCTGGAGAG 109

Nme GACACCCTTAAATCGCTCAGGTATCAGGGACTGCACATTGAAACAAAC-AATCTGGAGAG 109

Ngo GACACCCTTAAATCGCTCAGGTATCAGGGACTGCACATTGAAACAAAC-AATCTGGAGAG 109

Nsu GACACCCTTAAATCGCTCAGGTATCAGGGACTGCACATTGAAACAAAC-AATCTGGAGAG 109

Npo GATGCCCTTAAATCGCTCAGGCAACAGGGACTGCACATTGAAATAAAA-CATCTGGAGAG 108

Nfl GATGCCCTTAAATCGCTCAGGCAACAGGGACTGCACATTGAAATAAAA-CATCTGGAGAG 108

Nci GATGCCCTTAAATCGCTCAGGCAACAGGGACTGCACATTGAAATAAAA-CATCTGGAGAG 108

Nma GATGCCCTTAAATCGCTCAGGCAACAGGGACTGCACATTGAAATAAAA-CATCTGGAGAG 108

Nar GATACCCTTAAATCGCTCAGGTAAAAGG-ACTGCATATTGTCAACAAAAAATCTGGAGAG 104

Nca GACACCCTTAAATCGCTCAGGCAAAAGG-ACTGCATTT-ATCACAAA--CATCTGGAGAG 106

Nwa GACACCCTTAAATCGCTCAGGCAAAAGG-ACTGCATTT-ATCACAAA--CATCTGGAGAG 106

Nde GACACCCTTAAATCGCTCAGGCAAAAGG-ACTGTGCACACCATCACAAACATCTGGAGAG 107

Nwe GACACCCTTAAATCGCTCAGGTAAAAGG-ACTGCATTTCATCACAAAT-CATCTGGAGAG 109

Nano GACACCCTTAAATCGCTCAGGCAAAAGG-ACTGCATTTCATCAC-AAT-CATCTGGAGAG 117

Nzo GACACCCTTAAATCGCTCAGGCAAAAGG-ACTGCATTTCATCAC-AAT-CATCTGGAGAG 117

** ***************** * *** **** * **********

Nani CGGCTTGTGCCAC-CATAAGCCCACCGAAGGGGAAAAGGCAGTTTGAACCACCATTCAAA 169

Nmu CGGCGTTGGAA----TAACGTCCACCGAAGGGGAGAAGGCCGTCTGAACCACCATTCAGA 164

Nla CGGCGTTAGAA----TAACGTCCACCGAAGGGGAGAAGGCCGTCTGAACCACCATTCAGA 165

Nsi CGGCGTTGGAA----TAACGTCCACCGAAGGGGAGAAGGCCGTCTGAACCACCATTCAGA 165

Nme CGGCGTTGGAA----TAACGTCCACCGAAGGGGAGAAGGCCGTCTGAACCACCATTCAGA 165

Ngo CGGCGTTAGAA----TAACGTCCACCGAAGGGGAGAAGGCCGTCTGAACCACCATTCAGA 165

Nsu CGGCGTTAGAA----TAACGTCCACCGAAGGGGAGAAGGCCGTCTGAACCACCATTCAGA 165

Npo CGGCGTTTCCCAAAGATACGCCCACCGAAGGGGAGAAGGCCGTCTGAACCACCATTCAGA 168

Nfl CGGTGCGAT-------CGCACCCACCGAAGGGGAGAAGGCCGTCTGAACCACCATTCAGA 161

Nci TGGTGCAAT-------CGCACCCACCGAAGGGGAGAAGGCCGTCTGAACCACCATTCAGA 161

Nma CGGTGCAAT-------CGCACCCACCGAAGGGGAGAAGGCCGTCTGAACCACCATTCAGA 161

Nar TGGCGCGGC-------TGCGCCCACCGAAGGGGAGAAAGCCGTTT--------------- 142

Nca CGGCGCAGC-------AGTGCCCACCGAAGGGGAAAAGGC-GTTTGAATGCTTCAAACCG 158

Nwa CGGCGCAGC-------GGCGCCCACCGAAGGGGAAAAGGC-GTTTGAATGCTTCAAACCG 158

Nde CGGCGCAGC-------TGCGCCCACCGAAGGGGAGAAGGCCGTCTGAAAA--CCCAAGCC 158

Nwe CGGCGCGGC-------TGTGCCCACCGAAGGGAAAAAGGCCGTCTGAAAA--CATTCGGG 160

Nano CGGCGCGGC-------TGCGCCCACCGAAGGGGAGAAGGCCGTCTGAAAA--TATTCGGG 168

Nzo CGGCGCGGC-------TGCGCCCACCGAAGGGGAGAAGGCCGTCTGAAAA--CATTCGGG 168

** *********** * ** ** * *

Nani T--ACAGGCCGCGCCGCAAT-------------------------------GAGCAGA-T 195

Nmu C--AACCGCGCAAA-GCAGT-------------------------------GAGCAGACT 190

Nla C--AACCGCGCAAA-GCAGT-------------------------------GAGCAGACT 191

Nsi C--AACCGCGCAAA-GCAGT-------------------------------GAGCAGACT 191

Nme C--AACCGCGCAAA-GCAGT-------------------------------GAGCAGACT 191

Ngo C--AACCGCGCAAA-GCAGT-------------------------------GAGCAGACT 191

Nsu C--AACCGCGCAAA-GCAGT-------------------------------GAGCAGACT 191

Npo C--AACCGCGCAAA-GCAGT-------------------------------GAGCAGACT 194

Nfl C--AACCGCGCAAA-GCAGT-------------------------------GAGCAGACT 187

Nci C--AACCGCGCAAA-GCAGT-------------------------------GAGCAGACT 187

Nma C--AACCGCGCAAA-GCAGT-------------------------------GAGCAGACT 187

Nar ------------------------------------------------------------ 142

Nca ------------------------------------------------------------ 158

Nwa ------------------------------------------------------------ 158

Nde CGAAGCGGCGTCAAAACGGGTTCAGGCACACAAGCCGGTTTCAGACGGCCTGTGTTTCCG 218

Nwe CGGACACACAGGCATTCGATTCA------------------------------------- 183

Nano CGAACACACAGGCATTCGATTCA------------------------------------- 191

Nzo CGAACACACAGGCATTCGATTCA------------------------------------- 191

Nani ---TGCGGATAATGTGCGGTTTGCCGAAAATCTCAGGTTCAAGGACAGATAGGGTCGCCG 252

Nmu GGTTTGCCGTCCAGCGGATACGGCCGAAAATCTCAGGTTCAAGGACAGATAGGGTCATCC 250

Nla GGTTTGCCATCATGCGGATACGGCCGAAAATCTCAGGTTCAAGGACAGATAGGGTCATCC 251

Nsi GGTTTGCCATCATGCGGATACGGCCGAAAATCTCAGGTTCAAGGACAGATAGGGTCATCC 251

Nme GGTTTGCCATCATGCGGATACAGCCGAAAATCTCAGGTTCAAGGACAGATAGGGTCATCC 251

Ngo GGTTTGCCATCATGCGGATACGGTCGAAAATCTCAGGTTCAAGGACAGATAGGGTCATCC 251

Nsu GGTTTGCCATCATGCGGATACGGCCGAAAATCTCAGGTTCAAGGACAGATAGGGTCATCC 251

Npo GGTTTGCCATCATGCGGATACGGCCGAAAATCTCAGGTTCAAGGACAGATAGGGTCATCC 254

Nfl GGTTTGCCATCAAGCGGATACGGCCGAAAATCTCAGGTTCAAGGACAGATAGGGTCTTCC 247

Nci GGTTTGCCATCAAGCGGATACGGCCGAAAATCTCAGGTTCAAGGACAGATAGGGTCTTCC 247

Nma GGTTTGCCATCAAGCGGGTATG-CCGAAAATCTCAGGTTCAAGGACAGATAGGGTCTTCC 246

Nar ------------------TACGGCTGAAAATCTCAGGTATCAGGACAGATAGGGGCGCGT 184

Nca ----------------------GCCGAAAATCTCAGGTTAAAGGACAGATAGGGACGCGT 196

Nwa ----------------------GCCGAAAATCTCAGGTTAAAGGACAGATAGGGGCGCGT 196

Nde TTG--------CAACCGGAAAAGCCGAAAATCTCAGGTTCAAGGACAGATAGGGTCGCGT 270

Nwe -----------GACGGCCTGTTGCCGAAAATCTCAGGTTCAAGGACAGATAGGGGCGCGT 232

Nano -----------GACGGCCTGTTGCCGAAAATCTCAGGTTCAAGGACAGATAGGGGCGCGT 240

Nzo -----------GACGGCCTGTTGCCGAAAATCTCAGGTTCAAGGACAGATAGGGGCGCGT 240

************* *************

Nani CGCAGTCAAAGTGCGGAACGGCCTATAAATACCAAGTATCCGCCGCCATCGGATTTGCAG 312

Nmu GCGCACAGGTGCGCGGGCGGCATCT-GAACAATA----AATCCGGAGAAACTTGAGA**ATG** 305

Nla GCGCACAGGTGCGCGGGCGGCATCT-GAACAAAA----AATCCGGAGAAACTTGAGA**ATG** 306

Nsi GCGCATAGGTGCGCGGGCGGCATCT-GAACAAAA----AATCCGGAGAAACTTGAGA**ATG** 306

Nme GCGCACAGGTGCGCGGGCGGCATCT-GAACAAAA----AATCCGGAGAAACTTGAGA**ATG** 306

Ngo GCGCACAGGTGCGCGGGCGGCATCT-GAACAAAA----AATCCGGAGAAACTTGAGA**ATG** 306

Nsu GCGCACAGGTGCGCGGGCGGCATCT-GAACAAAA----AATCCGGAGAAACTTGAGA**ATG** 306

Npo GCGCACAGGTGCGCGGGCGGCATCT-GAACAAAA----AATCCGGAGAAACTTGAGA**ATG** 309

Nfl GCGCACAGGTGCGCGGGCGGCATCT-GAACAAAA----AATCCGGAGAAACTTGAGA**ATG** 302

Nci GCGCATCTTGT-GCGGACGACATCT-GAACAATA----AATCCGGAGAAGCT-GAGA**ATG** 300

Nma GCGCATCTTGT-GCGGACGGCATCT-GAACAATA----AATCCGGAGAAGCT-GAGA**ATG** 299

Nar GTAATCAAACGCGGCGGCCTGCTATGCTGTTTTTTCTTTATTGAAGTAAGAGCGTCCCAA 244

Nca GAAAAGCAACCGCACAGATGCGTTGCGGCCATTTCCCCCTATTCGGAGACCTGAAAC**ATG** 256

Nwa GAAAAGCAACCGCATAGATACGTTGCGGCCATTTCCCCCTATTCGGAGACCTGAAAC**ATG** 256

Nde GCAAAGCAAACGCCATCCT----------------TAACCTATGGAGAACC-GAGAC**ATG** 313

Nwe GAAAAGCATCGCAGCCCT-----------------TAACCTATGGAGAACCAGAGCA**ATG** 275

Nano GAAAAGCATCGCAGCCCT-----------------TAACCTATGGAGAACCAGAGTA**ATG** 283

Nzo GAAGAGCATCGCAGCCCT-----------------TAACCTATGGAGAACCAGAGCA**ATG** 283

**Fig. S6:**

Conservation of the *gcvT* tandem glycine riboswitch in the genus *Neisseria*. The upstream region of the *gcvT* gene from different *Neisseria* species comprising the riboswitch sequence was aligned using CLUSTAL omega (<https://www.ebi.ac.uk/jdispatcher/msa/clustalo>). Nucleotide segments forming the P1, P2 and P3 stems of aptamer 1 and 2 are color coded as follows: P1, grey; P2, yellow; P3, cyan. The *gcvT* start codon is marked in bold. Stars beneath the alignment indicate identical nucleotides at the respective position in all riboswitch sequences.

The three- or four-letter code identifying *Neisseria* species and strains used for the generation of the alignment is as follows (sequence ID is given in parentheses): Ngo: *N. gonorrhoeae* MS11 (CP003909.1); Nme: *N. meningitidis* MC58 ([AE002098.2](https://www.ncbi.nlm.nih.gov/nucleotide/AE002098.2?report=genbank&log$=nuclalign&blast_rank=1&RID=T1CYXMSG013)); Npo: *N. polysaccharea* M18661 ([CP031325.1](https://www.ncbi.nlm.nih.gov/nucleotide/CP031325.1?report=genbank&log$=nuclalign&blast_rank=1&RID=T1EHDZ6Y013)); Nci: *N. cinerea* NCTC10294 ([LS483369.1](https://www.ncbi.nlm.nih.gov/nucleotide/LS483369.1?report=genbank&log$=nuclalign&blast_rank=1&RID=T3FD86Z0016)); Nla: *N. lactamica* Y92-1009 ([CP019894.1](https://www.ncbi.nlm.nih.gov/nucleotide/CP019894.1?report=genbank&log$=nuclalign&blast_rank=1&RID=T1DD6CGR013)); Nani: *N. animalis* ATCC 49930 ([CP031699.1](https://www.ncbi.nlm.nih.gov/nucleotide/CP031699.1?report=genbank&log$=nuclalign&blast_rank=1&RID=T1DN1ETY013)); Nmu: *N. mucosa* FDAARGOS ([CP053939.1](https://www.ncbi.nlm.nih.gov/nucleotide/CP053939.1?report=genbank&log$=nuclalign&blast_rank=1&RID=T1DGEH8V016)); Nsi: *N. sicca* ATCC 29256 ([CP079820.1](https://www.ncbi.nlm.nih.gov/nucleotide/CP079820.1?report=genbank&log$=nuclalign&blast_rank=1&RID=T1DA0VJ4013)); Nfl: *N. flavescens* ATCC 13120 ([CP039886.1](https://www.ncbi.nlm.nih.gov/nucleotide/CP039886.1?report=genbank&log$=nuclalign&blast_rank=1&RID=T3G0R8N8013)); Nsu: *N. subflava* TT0077 ([CP073115.1](https://www.ncbi.nlm.nih.gov/nucleotide/CP073115.1?report=genbank&log$=nuclalign&blast_rank=1&RID=T1D5G68U016)); Nde: *N. dentiae* DSM 19151 ([CP059570.1](https://www.ncbi.nlm.nih.gov/nucleotide/CP059570.1?report=genbank&log$=nuclalign&blast_rank=1&RID=T3HKP25C013)); Nma: *N. macacae* ATCC 33926 ([CP094241.1](https://www.ncbi.nlm.nih.gov/nucleotide/CP094241.1?report=genbank&log$=nuclalign&blast_rank=1&RID=T3H176UC01N)); Nano: *N. animaloris* NCTC12227 ([LR134516.1](https://www.ncbi.nlm.nih.gov/nucleotide/LR134516.1?report=genbank&log$=nuclalign&blast_rank=1&RID=T3EX67Y0013)); Nzo: *N. zoodegmatis* NCTC12230 ([LT906434.1](https://www.ncbi.nlm.nih.gov/nucleotide/LT906434.1?report=genbank&log$=nuclalign&blast_rank=1&RID=T3G6ZSAZ016)); Nwe: *N. weaveri* NCTC12742 ([LR134533.1](https://www.ncbi.nlm.nih.gov/nucleotide/LR134533.1?report=genbank&log$=nuclalign&blast_rank=1&RID=T3FK29FB016)); Nca: *N*. *canis* NCTC10296 ([LR134313.1](https://www.ncbi.nlm.nih.gov/nucleotide/LR134313.1?report=genbank&log$=nuclalign&blast_rank=1&RID=T3GF9PYS01N)); Nwa: *N. wadsworthii* DSM 22245 ([CP059565.1](https://www.ncbi.nlm.nih.gov/nucleotide/CP059565.1?report=genbank&log$=nuclalign&blast_rank=1&RID=T3GUAD4V01N)); Nar: *N*. *arctica* KH1503 ([CP091510.1](https://www.ncbi.nlm.nih.gov/nucleotide/CP091510.1?report=genbank&log$=nuclalign&blast_rank=1&RID=T3K0VMVR01N))


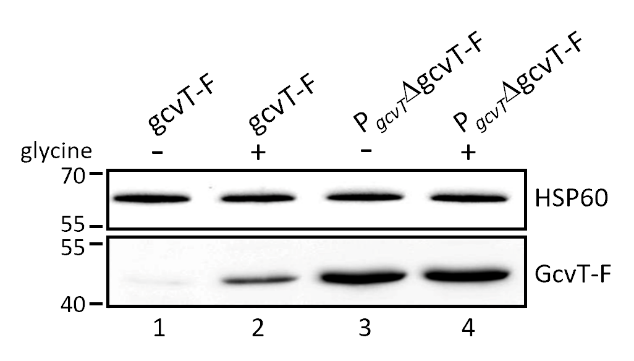


**Fig. S7:**

Immunoblot analysis of *N. gonorrhoeae* MS11 gcvT-F and MS11 P*_gcvT_*ΔgcvT-F with truncated *gcvT* 5’-UTR. Gonococci were grown in CDM10 medium containing no (lane 1 and 3) or 2.5 mM (lane 2 and 4) glycine to OD_550_ = 0.4. Bacteria were lysed and equal amounts of proteins were analysed by Western Blot using monoclonal antibodies directed against the 3xFLAG epitope and HSP60 used as loading control. Protein samples for detection of GcvT-F and HSP60 by immunoblotting were run on separate gels. The figure shows the results from a representative experiment (n = 3). Numbers on the left side of the panel indicate the position of size marker proteins [kDa].


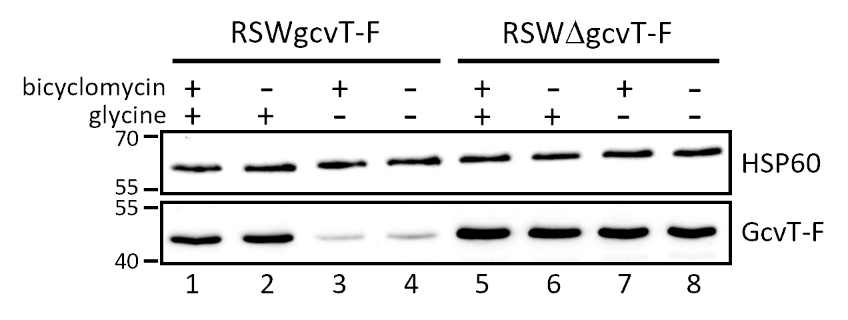


**Fig. S8:**

Glycine-responsive expression of 3xFLAG-tagged *gcvT* in *N. gonorrhoeae* strains MS11 RSWgcvT-F and MS11 RSWΔgcvT-F in the absence and presence of bicyclomycin. Strains RSWgcvT-F (lanes 1 to 4) and RSWΔgcvT-F (lanes 5 to 8) were cultivated to OD_550_ = 0.4. in CDM10 medium in the presence (2.5 mM; lanes 1, 2, 5 and 6) or absence of glycine (lanes 3, 4, 7 and 8) and presence (10 mM, lanes 1, 3, 5 and 7) or absence of bicyclomycin (lanes 2, 4, 6 and 8). Equal amounts of protein from lysed bacteria were separated on 10% SDS polyacrylamide gels. Protein samples for detection of GcvT-F and HSP60 by immunoblotting were run on separate gels. The results from a representative experiment are shown (n =2). Numbers on the left side of the panel indicate the position of size marker proteins [kDa].
